# Identification of the immunoprotective activity of Mori Cortex Radicis ethanol extract against aquaculture pathogens (*Aeromonas veronii*, *Pseudomonas aeruginosa*)

**DOI:** 10.3389/fimmu.2026.1927434

**Published:** 2026-08-17

**Authors:** Xing Zhang, Ling Zhu, Yixin Yu, Kai Wang, Qinkai Hu, Xuan Huang, Guangbao Zhu, Jingjing Ma, Shanshan Li, Xiang Liu

**Affiliations:** 1 Anhui Province Key Laboratory of Embryo Development and Reproductive Regulation, Fuyang Normal University, Fuyang, China; 2 Rural Revitalization Collaborative Technology Service Center of Anhui Province, Fuyang Normal University, Fuyang, China

**Keywords:** *Aeromonas veronii*, ethanol extract, immune protection, LC-MS, Mori Cortex Radicis, *Pseudomonas aeruginosa*

## Abstract

**Objective:**

Mori Cortex Radicis (MCR) is an important Chinese herbal medicine with antitussive, anti-inflammatory, and analgesic effects. It is abundant in resources and low in cost, but its application in the control of fish pathogens has been insufficiently studied.

**Methods:**

We employed an ethanol extraction method to obtain the MCR ethanol extract (MCR-EE), and analyzed its small-molecule and macromolecular components using LC-MS and commercial kits, respectively. Subsequently, using a crucian carp (*Carassius auratus*) model fed with MCR-EE and challenged with aquaculture pathogens, the immunoprotective activities of MCR-EE were evaluated through a combination of survival rate monitoring, leukocyte phagocytosis assays, immune factor activities, qRT-PCR detection of mRNA expression of inflammatory cytokines and antioxidant pathway factors, histopathological examination of visceral tissues, and immunofluorescence analysis of the expression of the apoptotic factor (p53) and the DNA damage marker (γH2AX).

**Results:**

The MCR-EE was prepared. The contents of proteins, polysaccharides, and polyphenols of MCR-EE were 0.7%, 0.44%, and 1.21%, respectively. A total of 646 small substances were identified in MCR-EE, and the five most abundant small-molecule compounds were L(+)-Arginine, 1-Deoxynojirimycin, 9,12,13-Todea, 4-Guanidinobutanoic acid, and Kelampayoside A. The MCR-EE enhanced the general phagocytic activity of fish blood leukocytes and the activity of immune factors. The immune protection rates of MCR-EE against *Aeromonas veronii* and *Pseudomonas aeruginosa* were 46.67% and 40% (*p* < 0.05), respectively. MCR-EE reduced the bacterial loads of *A. veronii* and *P. aeruginosa* in kidney tissues, as well as the mRNA expression of inflammatory cytokines (IL-6, TNF-α, and IL-1β) and key factors of oxidative stress pathway (Nrf2, HO-1, Keap1) (*p* < 0.05), and decreased the levels of antioxidant factors (*p* < 0.05). Histopathological examination revealed that MCR-EE maintained the structural integrity of the spleen, kidney, and intestine, and decreased the expression of p53 and γH2AX (*p* < 0.05) in kidney.

**Conclusion:**

MCR-EE exhibits immunomodulatory activity and holds promise as an immunostimulant for the prevention and control of fish pathogens (*A. veronii* and *P. aeruginosa*).

## 1 Introduction

Aquaculture, as a key industry providing high-quality protein and ensuring food security, has been experiencing rapid and sustained global development in recent years (1). However, under intensive, high-density farming conditions, the issue of pathogenic bacterial infections has become increasingly prominent, posing a critical bottleneck to the healthy and sustainable development of the industry (2, 3). Common aquatic pathogenic bacteria include *Aeromonas veronii*, *Pseudomonas aeruginosa*, *Vibrio parahaemolyticus*, *Vibrio harveyi*, *Aeromonas hydrophila*, and *Edwardsiella tarda* (4–6), which can cause severe diseases such as septicemia, ulcers, and hemorrhagic ascites, leading to large-scale mortality of farmed animals and significant economic losses (7). Currently, antibiotics remain the primary means of controlling such bacterial diseases, mainly including neomycin sulfate, doxycycline hydrochloride, enrofloxacin, and ciprofloxacin hydrochloride (8, 9). Nevertheless, their long-term and improper use has led to the continuous emergence of drug-resistant strains, increased environmental and ecological risks, and issues of drug residues in aquatic products, seriously hindering the sustainable development of aquaculture (10, 11). Therefore, the development of safe, efficient, and environmentally friendly antibiotic alternatives has become a research priority in the field of fish disease prevention and control.

Natural medicinal plant extracts are rich in various active components such as flavonoids, alkaloids, phenols, terpenoids, polysaccharides, and volatile oils, and possess broad-spectrum antibacterial, immunomodulatory, antioxidant, and anti-inflammatory effects (12, 13). Compared with antibiotics, plant extracts are less likely to induce bacterial resistance, can be metabolized and degraded *in vivo*, have low environmental impact, and are widely available with relatively controllable costs (14, 15). Studies have shown that extracts of *Urtica magellanica*, *Curcuma* spp., and lavender enhance immune responses in fish and reduce mortality caused by *A. hydrophila* infection (16–18). Quercetin extract exhibits significant antibacterial effects against *V. parahaemolyticus* and *Vibrio alginolyticus* (19). Dietary supplementation with papaya (*Carica papaya*) leaf extract in carp significantly improves growth performance, intestinal enzyme activity, skin mucosal immune parameters, and immunity (20). The combination of *Scutellaria baicalensis*, *Forsythia suspensa*, and *Rheum palmatum*, as well as compound plant extracts from *Eucalyptus globulus* and *Psidium guajava*, show good *in vitro* antibacterial activity against *V. parahaemolyticus*, and dietary supplementation significantly improves fish survival rates (21, 22). Thus, various plant extracts can effectively inhibit the growth of fish pathogenic bacteria, reduce infection mortality, and enhance both non-specific and specific immune responses in fish, demonstrating promising application prospects. However, given the vast diversity of natural plants, further screening and identification are needed to evaluate the immunomodulatory activity of their extracts in fish.

Mori Cortex Radicis (MCR) is the dried root bark of *Morus alba* L., a plant of the Moraceae family. It is widely cultivated in China, with abundant resources and low cost (23). In traditional Chinese medicine, the MCR is believed to have the effects of antitussive, as well as promoting diuresis to reduce edema (24, 25). Pharmacological studies have shown that the MCR contains various active components such as flavonoids, alkaloids, terpenoids, and phenols, exhibiting good antioxidant, anti-inflammatory, hypoglycemic, hypolipidemic, and antibacterial activities (26–28). Hong et al. found that MCR inhibits osteoclast differentiation, fusion, and the expression of bone resorption markers by suppressing the expression of NFATc1/c-Fos, thereby exerting a therapeutic effect on osteoporosis (29). In mice, combined administration of MCR and Mori Folium was found to regulate adipocyte metabolic disorders through the AMPK signaling pathway, providing a functional food for the prevention of obesity and related metabolic diseases (30). Unlike many tropical medicinal plants (e.g., turmeric, moringa) that are limited to specific climatic zones, MCR is widely cultivated across temperate and subtropical regions of China, ensuring a consistent and sustainable raw material supply regardless of seasonal or geographic variation (23). Moreover, MCR has a long history of use in traditional Chinese medicine for treating coughs, edema, and inflammatory conditions, with no significant toxicity reported in long-term applications (24, 25). This established safety record is of particular importance for its use in food-producing animals, including fish, where concerns regarding consumer safety and drug residues are critical. Despite these advantages and the well-documented pharmacological activities of MCR in mammals, its application in aquaculture, particularly as an immunostimulant against bacterial diseases in fish, remains largely unexplored. This significant knowledge gap, combined with its abundant availability, low cost, compositional diversity, and established safety, provides a strong rationale for evaluating MCR as a novel and cost-effective immunostimulant for aquaculture.

**Figure 1:**
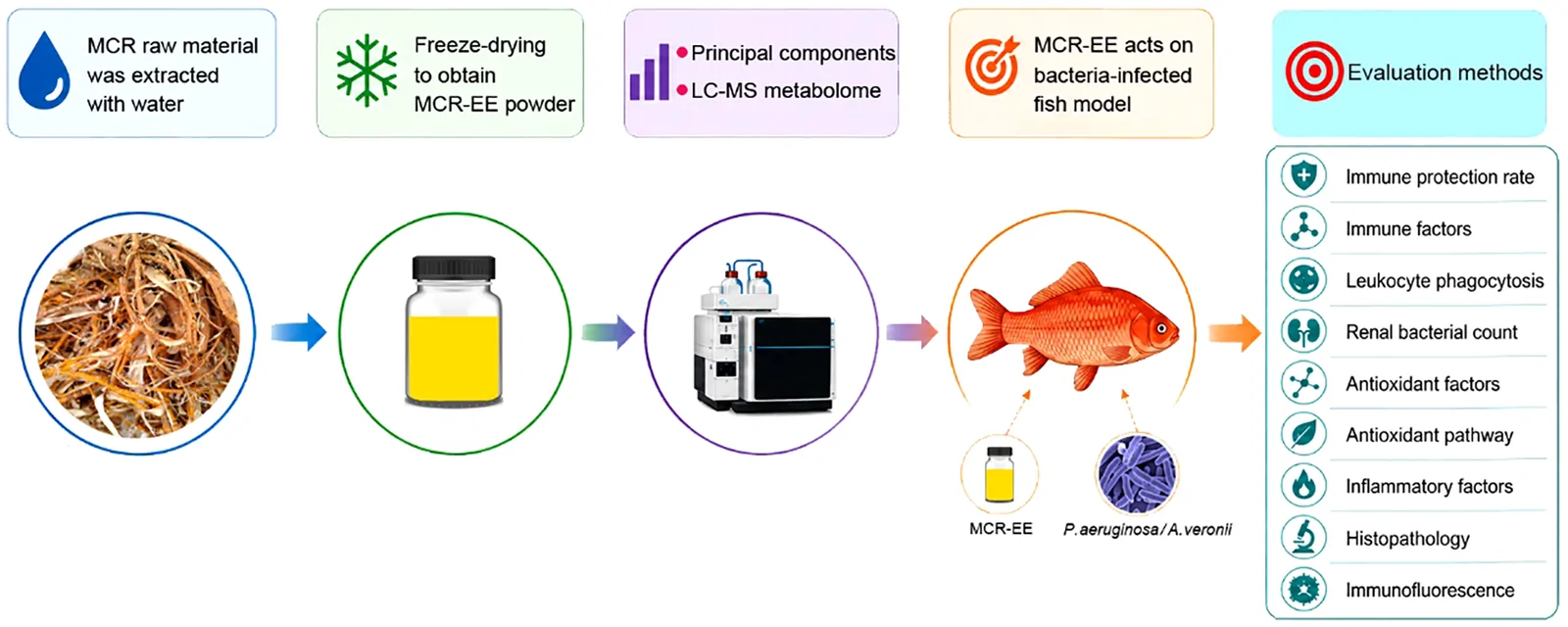
Experimental design.

This study focused on the extraction of the MCR ethanol extract (MCR-EE) and the analysis of its chemical composition. Using a crucian carp (*Carassius auratus*) model fed with MCR-EE and challenged with aquatic bacterial pathogens (*A. veronii* and *P. aeruginosa*), the immunomodulatory activity of MCR-EE was evaluated through molecular biological approaches (**Figure 1**). This study aims to lay a foundation for the application of MCR in the prevention and control of bacterial pathogens in aquaculture.

## 2 Materials and methods

### 2.1 Strains and animals

MCR was purchased from a Chinese herbal medicine trading market (Bozhou, China). *A. veronii*, *P. aeruginosa*, and *Staphylococcus aureus* strains were preserved by the Molecular Laboratory of Fuyang Normal University. Crucian carp (25 ± 1 g) were purchased from Fuyang Aquaculture Co., Ltd. (Fuyang, China). All animal experiments were conducted in accordance with the Guide for the Care and Use of Laboratory Animals and were approved by the Institutional Animal Care and Use Committee of Fuyang Normal University, China (No. 2025-07-016, July 8, 2025).

### 2.2 Preparation of MCR ethanol extract and analysis of biomacromolecule contents

The MCR-EE was prepared following a previously published method (31). Briefly, 1.5 kg of MCR was freeze-dried to complete dehydration, pulverized using a high-speed blender, and sieved through an 80-mesh screen. The MCR powder was soaked and stirred in 70% ethanol at a solid-to-liquid ratio of 1:15 (m/v), then refluxed at 60 °C for 3 h. The mixture was filtered, and the filtrate was collected. The residue was subjected to the same procedure, and the two filtrates were combined. Then, the filtrate was rotary-evaporated at 50 °C and 100 r/min to 1/20 of the original volume, followed by drying in a freeze dryer to obtain MC-EE powder. In addition, the contents of proteins, polysaccharides, and polyphenols in the MCR-EE were determined using commercial kits according to the manufacturer’s instructions (Servicebio Technology Co., Ltd., Wuhan, China).

### 2.3 LC-MS analysis of small molecule compounds in MCR-EE

Liquid chromatography-mass spectrometry (LC-MS) was performed to detect small molecule compounds in MCR-EE according to previously method (32). Briefly, 50 mg of MCR-EE was placed into a 2 mL centrifuge tube, along with one 6 mm diameter grinding bead. Then, 400 μL of extraction solution (methanol: water = 4: 1, v/v) was added. The sample was ground for 6 min using a frozen tissue grinder, followed by low-temperature ultrasonic extraction for 30 min. After extraction, the sample was centrifuged at 13, 000 g and 4 °C for 15 min, and the supernatant was collected. LC-MS analysis was carried out on a Thermo Fisher UHPLC-Q Exactive system (Thermo Fisher Scientific, Waltham, MA, USA) coupled with Fourier transform mass spectrometry. The chromatographic column was an ACQUITY UPLC BEH C18 (Waters, Milford, USA). Mobile phase A and B were 2% acetonitrile (containing 0.1% formic acid) and acetonitrile (containing 0.1% formic acid), respectively. MCR-EE were electrospray ionized, and mass spectra were acquired in both positive and negative ion scanning modes. The scan type (m/z) parameter was 70–1050; sheath gas flow rate (arb) was 50; auxiliary gas flow rate (arb) was 13; capillary temperature was 320 °C; heater temperature was 450 °C; spray voltage (+) was 3500 V; spray voltage (–) was –3000 V. A mass spectrum was obtained with each scan, and the total ion current intensity was obtained by summing the intensities of all ions in the mass spectrum. Blank samples were prepared by substituting MCR-EE powder with the extraction solvent (methanol: water = 4:1, v/v), and the complete extraction and injection procedures were carried out to identify and subtract background interference signals introduced by the solvents and mobile phase. All sample data were background-corrected by subtracting the blank signal prior to subsequent analysis. For quality-control (QC) samples, the instrument performance was considered acceptable when the relative standard deviation (RSD) of retention times for major characteristic peaks was < 2% and the RSD of peak areas was < 30%. Subsequently, the raw data were imported into the metabolomics processing software Progenesis QI v3.0 (Waters, Milford, USA) for peak identification, baseline filtering, integration, retention time correction, and peak alignment, resulting in a data matrix containing information on retention time, mass-to-charge ratio, and peak intensity. Then, the acquired MS and MS/MS spectra were matched against a proprietary traditional Chinese medicine metabolite database (Majorbio Bio-pharm Technology Co., Ltd., Shanghai, China) with a mass tolerance threshold of < 10 ppm. Meanwhile, the metabolites were subjected to accurate matching against a publicly available computationally simulated MS/MS library (public and theoretical databases), and only compounds with a match score > 35 were retained for cross-validation of the MS/MS spectral assignments. Final identifications were retained only for those compounds that showed consistent matches in both independent databases, thereby ensuring the reliability of the identifications. The resulting compounds were then ranked according to their relative MS signal matching scores.

### 2.4 Evaluation of the immune protection rate of MCR-EE

Crucian carp (25 ± 1) were allocated into two main groups for challenge with *A. veronii* and *P. aeruginosa*, respectively. Each main group was subdivided into two subgroups (15 fish each), yielding four subgroups in total. Each group (15 fish) was housed in one 1000-L tank. The tanks were randomly assigned to positions on the racks within the same tier, ensuring that light intensity and incident angle were uniform across all tanks. Fish were randomly selected from the acclimation pool and sequentially allocated to designated aquaria. The fish farming laboratory temperature was set at 22 °C with a relative humidity of 60%–80%, and the water pH was maintained at approximately 7.0. Each tank was equipped with an aeration and filtration system, and one-quarter of the water was renewed daily. All experimental procedures, including treatment administration, mortality monitoring, tissue collection, and laboratory assays, were performed by researchers blinded to group allocation. The blinding code was broken only after completion of data acquisition. Fish in the control group (Ctr) received 40 μL of sterile water by oral gavage using a flexible plastic feeding tube attached to a micro-syringe, while the experimental group were given 40 μL of MCR-EE (200 mg/kg body weight/day) dissolved in sterile water at a concentration of 125 μg/μL. Feeding was performed once daily for seven consecutive days. Based on the median lethal dose (LD_50_) of bacterial challenge in fish determined in preliminary experiments, intraperitoneal challenge was then conducted using *A. veronii* (1.0 × 10^9^ CFU) or *P. aeruginosa* (1.5 × 10^9^ CFU) in crucian carp. Mortality was monitored for 14 consecutive days. The relative percent survival (RPS) was calculated as: RPS (%) = (1 − [mortality of MCR-EE group (%)/mortality of the Ctr group (%)]) × 100. Statistical significance was analyzed using SPSS 20.0 software (33).

### 2.5 Detection of immune factors in crucian carp serum

Crucian carp were divided into two groups, with six fish per group. The control group (Ctr) was administered 40 μL of sterile water, while the MCR-EE group received 40 μL of MCR-EE (200 mg/kg body weight/day). After seven consecutive days of feeding, the fish were anesthetized with tricaine methanesulfonate (MS-222), and blood was collected from the caudal vein. The blood samples were placed in a refrigerator at 4 °C overnight. Subsequently, the samples were centrifuged at 3000 r/min for 20 min at 4 °C, and the supernatants were collected to obtain fish serum. The activities of acid phosphatase (ACP), alkaline phosphatase (AKP), and lysozyme (LZM) were measured according to the kit manufacturer’s instructions (Servicebio Technology Co., Ltd., Wuhan, China).

### 2.6 Detection of general leukocyte phagocytosis in fish blood

Fish were divided into two groups, with six fish per group. They were fed sterile water or MCR-EE for 7 consecutive days, respectively. After 7 days, the fish were anesthetized, and blood was collected from the caudal vein using anticoagulant-containing centrifuge tubes. Meanwhile, cultured *S. aureus* was inactivated with 1% formaldehyde in an 80 °C water bath for 1 hour. Then, 0.3 mL of blood was mixed with 0.3 mL of inactivated *S. aureus* (2 × 10^8^ CFU) and incubated in a 25 °C water bath for 60 minutes. *S. aureus* possesses a round shape for convenient counting and has been widely used in the analysis of leukocyte phagocytosis (33, 34). Subsequently, a small amount of the mixture was evenly smeared onto glass slides using a rubber-tipped dropper to prepare blood smears. The blood smears were stained with Giemsa stain (Servicebio, Wuhan, China). Finally, the phagocytic cells were counted under a microscope. The phagocytic percentage (*PP* %) was calculated as: (number of phagocytic cells among 100 phagocytes/100) × 100%. The phagocytic index (*PI* %) was calculated as: (number of bacteria inside phagocytes/number of phagocytic cells involved in phagocytosis) × 100%. Data were statistically analyzed using SPSS 20.0 software (33).

### 2.7 Bacterial count in kidney

Fish were divided into two major groups for challenge with *A. veronii* and *P. aeruginosa*, respectively. Each major group was further divided into two subgroups, resulting in a total of four subgroups, with six fish per subgroup. The control group was fed 40 μL of sterile water, while the MCR-EE group was fed 40 μL of MCR-EE (200 mg/kg body weight/day). In addition, a negative control group (6 fish) was set up, comprising fish that received neither the immunizing agent nor the bacterial challenge. The fish were fed once a day. After 7 consecutive days of feeding, they were intraperitoneally challenged with *A. veronii* (0.5 × 10^9^ CFU) and *P. aeruginosa* (0.75 × 10^9^ CFU), respectively. Two days after bacterial challenge, kidney tissues were collected. A 40 mg portion of kidney tissue was weighed and homogenized in 300 μL of physiological saline. Finally, the kidney homogenate was evenly spread onto LB agar plates and cultured overnight at 30°C in an incubator. The number of bacterial colonies on the plates was counted, and the clearance effect of MCR-EE on *A. veronii* and *P. aeruginosa* in crucian carp was evaluated based on the colony counts) (34).

### 2.8 Antioxidant factor analysis

Crucian carp were divided into two major groups for challenge with *A. veronii* and *P. aeruginosa*, respectively. Each major group was further divided into two subgroups, with six fish per subgroup. The control group was fed 40 μL of sterile water, and the experimental group was fed 40 μL of MCR-EE (200 mg/kg body weight/day). After 7 consecutive days of feeding, the fish were challenged with *A. veronii* (0.5 × 10^9^ CFU) and *P. aeruginosa* (0.75 × 10^9^ CFU), respectively. Two days later, blood was obtained from the caudal vein of the fish and placed in a refrigerator at 4 °C overnight. Subsequently, the samples were centrifuged at 3000 r/min for 20 min at 4 °C, and the supernatant was collected to obtain fish serum. The activities of catalase (CAT), glutathione peroxidase (GSH-Px), and superoxide dismutase (SOD), as well as the content of malondialdehyde (MDA), were measured using commercial kits (Servicebio Technology Co., Ltd., Wuhan, China). Data were statistically analyzed using SPSS 20.0 software (34).

### 2.9 mRNA expression analysis of inflammatory factors and antioxidant pathway-related factors

The mRNA expression of inflammatory factors and antioxidant pathway-related factors were evaluated using real-time quantitative PCR (qRT-PCR) according to previous experimental methods (35). Briefly, crucian carp were divided into two major groups for challenge with *A. veronii* and *P. aeruginosa*, respectively. Each major group was further divided into two subgroups, with nine fish per subgroup. The control group was fed 40 μL of sterile water, and the experimental group was fed 40 μL of MCR-EE (200 mg/kg body weight/day). After 7 days of feeding, the fish were challenged with *A. veronii* (0.5 × 10^9^ CFU) and *P. aeruginosa* (0.75 × 10^9^ CFU), respectively. Two days after bacterial challenge, kidney, spleen, and liver tissues were collected from both the experimental and control groups. Kidneys, spleens, and livers from three fish per group were pooled together, yielding three pooled samples per group (n = 3). Subsequently, the tissues were mixed with 2 mL of Trizol reagent (RNA inhibitor and lysate) and immediately homogenized using a portable tissue grinder to prevent RNA degradation. Total RNA was extracted according to the instructions of the RNA extraction kit (Takara, Dalian, China), and reverse transcribed into cDNA according to the instructions of reverse transcription kit (Takara, Dalian, China). qRT-PCR was performed using the SYBR^®^ Green Premix kit (Takara, Dalian, China) and synthesized primers (**Supplementary Table 1**). The ΔCt (cycle threshold change) values of the experimental and Ctr group were obtained by comparing the Ct value of the target gene with that of the reference gene (*gapdh*). The ΔΔCt value was then calculated by comparing the ΔCt values between the experimental and Ctr group. Subsequently, the mRNA expression of inflammatory factors was analyzed using the 2^-ΔΔCt^ formula. Finally, data were statistically analyzed using SPSS 20.0 software.

### 2.10 Histopathological analysis

Histopathological section observation was performed according to previous methods (34). Briefly, crucian carp were divided into two major groups for challenge with *A. veronii* and *P. aeruginosa*, respectively. Each major group was further divided into two subgroups, with six fish per subgroup. The control group was fed 40 μL of sterile water, and the experimental group was fed 40 μL of MCR-EE (200 mg/kg body weight/day). After 7 consecutive days of feeding, the fish were challenged with *A. veronii* and *P. aeruginosa*, respectively. Two days later, kidney, spleen, and intestinal tissues were collected and fixed by immersion in Davidson’s fixative and 10% formaldehyde solution for 24 hours, respectively. Subsequently, the tissues were dehydrated in a graded ethanol series (70, 80, 90, 95, and 100% ethanol), followed by clearing twice with xylene. Then, the tissues were embedded in paraffin at 60 °C and cut into 5 μm thick sections using a paraffin microtome. The sections were placed on glass slides, dried on a slide warmer at 60 °C for 3 hours, deparaffinized with xylene and rehydrated through a graded ethanol series. Next, the sections were stained with hematoxylin for 1 minute, rinsed with water, and then stained with eosin for 5 minutes. Finally, the sections were cleared with xylene and mounted with neutral resin, and observed and photographed under a microscope. The protective effect of MCR-EE on the organ tissue structure of the fish was analyzed based on the damage in the histopathological sections.

Histopathological damage was evaluated using a double-blind semi-quantitative scoring system. Three random fields of view per section were examined at 200 × magnification. Sections were independently reviewed by two trained pathologists who were blinded to the group allocation. A semi-quantitative scoring system (0–4 scale) was used to quantify tissue damage in the kidney, spleen, and intestine. (1) The scoring criteria for the kidney were based on tubular epithelial cell degeneration, glomerular structure, and interstitial inflammatory cell infiltration, as follows: 0 points, normal tissue architecture with no visible lesions; 1 point (mild), < 25% of the tissue area showing pathological changes; 2 points (moderate), 25–50% of the tissue area showing pathological changes; 3 points (severe), 50–75% of the tissue area showing pathological changes; and 4 points (very severe), > 75% of the tissue area showing pathological changes. (2) The scoring criteria for the spleen were based on the structural integrity of the white pulp, the degree of red pulp congestion, and nuclear density: 0 points, normal tissue architecture with clearly discernible white pulp; scores of 1–4 points were assigned according to the same grading criteria as described for the kidney. (3) The scoring criteria for the intestine were based on mucosal epithelial integrity, villus structure, and inflammatory cell infiltration: 0 points, intact villi with well-arranged epithelial cells; scores of 1–4 points were assigned according to the same grading criteria as described for the kidney (34).

### 2.11 Immunofluorescence analysis of fish kidney

Immunofluorescence analysis of kidney sections was performed according to previous experimental methods (35). Briefly, kidney tissue sections were deparaffinized in xylene and rehydrated through a graded ethanol series. The sections were treated with antigen retrieval solution. Subsequently, a circle was drawn around the kidney tissue sections using an immunohistochemistry pen, and 40 μL of 5% bovine serum albumin blocking solution was added to the tissue sections, followed by blocking overnight at 4 °C. After three washes with PBST solution, monoclonal antibodies against p53 or γH2AX (1:1000) were added to the tissues and incubated for 1 hour at 37 °C. Following washing with PBST solution, secondary antibody solution (1: 1500) was added and incubated for 1 hour at 37 °C. Under light-protected conditions, the cell nuclei were stained with 4′,6-diamidino-2-phenylindole (DAPI) for 10 minutes. Images were captured using a fluorescence microscope. Four fish were included in each group, and one kidney section was obtained from each fish. Sections were first examined under low-power magnification (×100) to assess overall tissue morphology and to exclude areas with obvious necrosis or severe processing artifacts. One random field per section was then selected at ×400 magnification to ensure representativeness, yielding a total of four images per group (n = 4). All images were acquired using identical exposure time, gain, and fluorescence intensity threshold settings. Image analysis was performed using ImageJ 1.54g software. The fluorescence intensities of p53 and γH2AX were normalized to the DAPI nuclear counterstain (target protein/DAPI ratio) to correct for variations in section thickness and cell density. Finally, statistical significance analysis of the data was performed using SPSS 20.0 software.

### 2.12 The statistical analysis

All experiments were performed in triplicate. The verification of data normality was assessed by Shapiro-Wilk test. Significant differences between the experimental group and control group were evaluated using SPSS 20.0 software with one-way analysis of variance (ANOVA) followed by Tukey’s multiple comparison test or independent samples t-test/Chi-square test. Results were expressed as mean ± standard deviation (SD). *p* < 0.05 was considered statistically significant (34).

## 3 Results

### 3.1 Principal component analysis of MCR-EE

The main components of MCR-EE were analyzed. The results showed that the contents of proteins, polysaccharides, and polyphenols were 0.70%, 0.44%, and 1.21%, respectively (**Table 1**).

**Table 1:** Main components of MCR-EE.

No.	Composition	Content ratio (%)
1	Proteins	0.70 ± 0.03
2	Polysaccharides	0.44 ± 0.01
3	Polyphenols	1.21 ± 0.02

### 3.2 Detection of small molecule substances in MCR-EE by LC-MS

**Figure 2:**
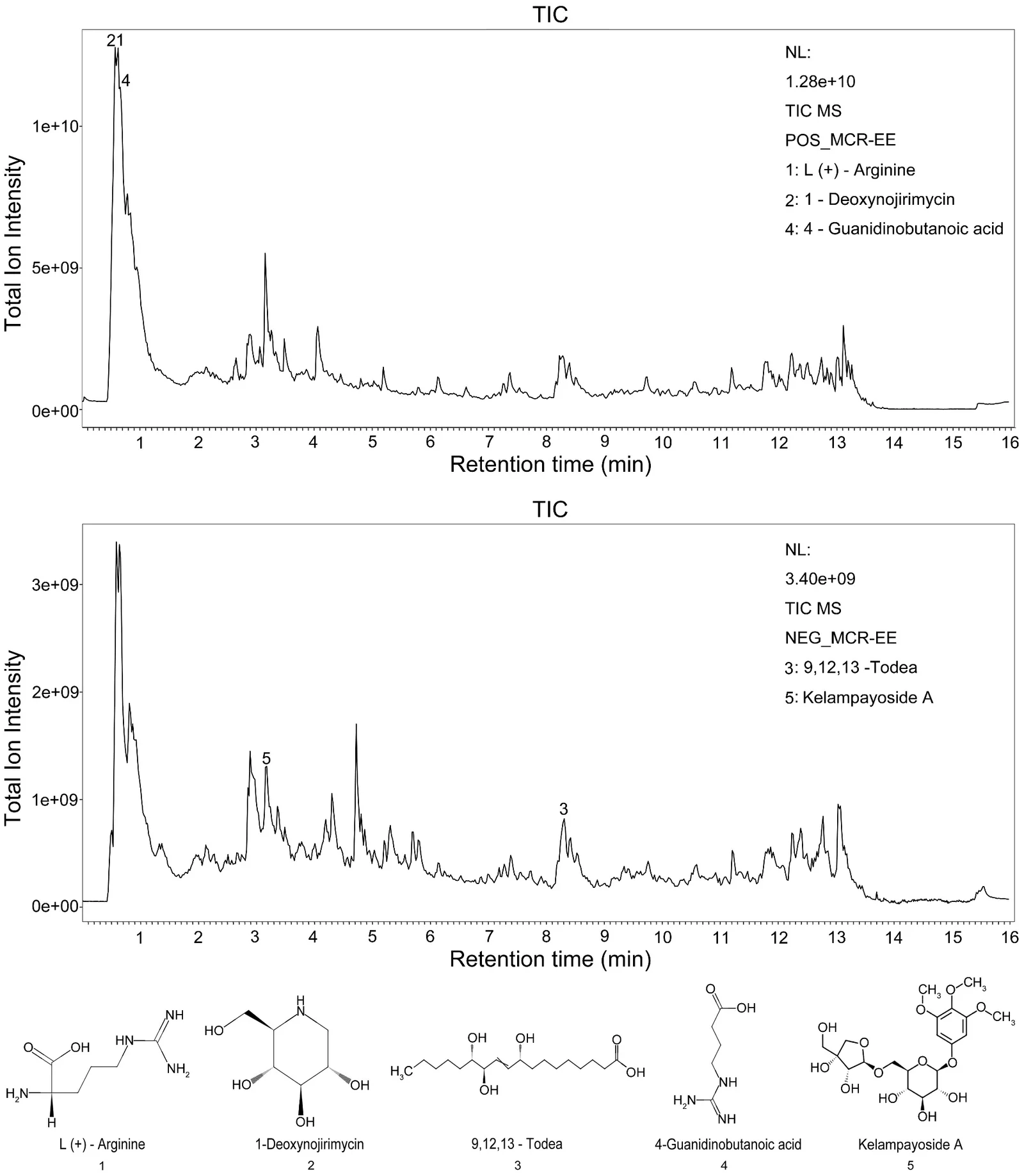
LC-MS metabolomics of MCR-EE.

Quantitative detection of small molecule substances in MCR-EE was performed using LC-MS. A total of 646 small molecule substances were identified (**Supplementary Table 2**). The LC-MS metabolomics data of MCR-EE are shown in **Figure 2**. Five small molecule substances were found to have relatively high contents, namely L(+)-Arginine, 1-Deoxynojirimycin, 9,12,13-Todea, 4-Guanidinobutanoic acid, and Kelampayoside A (**Table 2**).

**Table 2:** The 5 small-molecule substances with the highest contents in MCR-EE.

No.	Metabolite	Formula	M/Z	Retention time/min	Fragmentation score	Mode	Abundance
1	L(+)-Arginine	C6H14N4O2	175.1188	0.5607	93.2	pos	21031937.000
2	1-Deoxynojirimycin	C6H13NO4	164.0916	0.5252	96	pos	9844135.342
3	9,12,13-Todea	C18H34O5	329.233	8.3127	91.3	neg	9774943.176
4	4-Guanidinobutanoic acid	C5H11N3O2	146.0922	0.6321	94.7	pos	6862460.962
5	Kelampayoside A	C20H30O13	477.1608	3.1636	79.5	neg	6511341.679

### 3.3 Detection of serum immune factor activity in crucian carp

**Figure 3:**
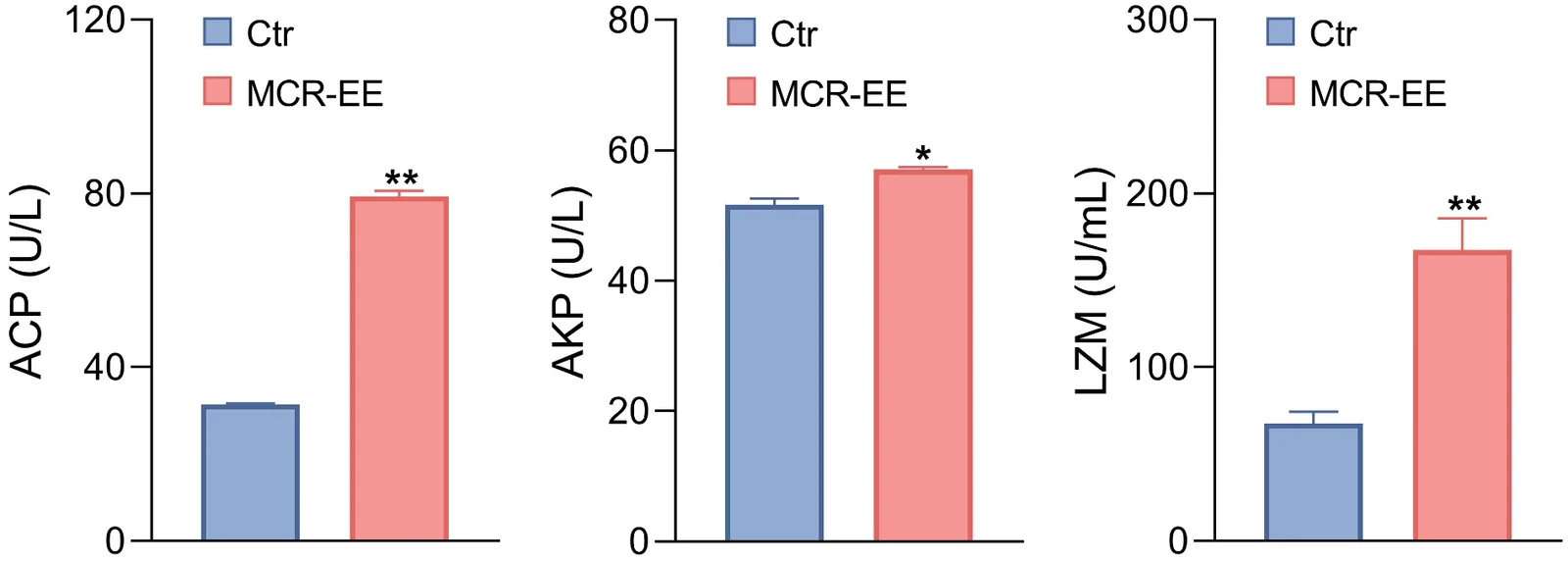
Analysis of serum immune factor activities. ^*^p < 0.05, ^**^p < 0.01 (compared with control).

After feeding crucian carp with MCR-EE, the activities of immune factors (ACP, AKP, LZM) in the serum showed a significant increasing trend (*p* < 0.05) (**Figure 3**). This indicates that MCR-EE can activate the immune response of crucian carp.

### 3.4 General phagocytic capacity of blood leukocytes in crucian carp

After feeding crucian carp with MCR-EE for 7 days, fish blood was collected for leukocyte phagocytic activity analysis. The results showed that the phagocytic index and phagocytic percentage of leukocytes in the MCR-EE group were significantly increased (*p* < 0.05) (**Table 3**).

**Table 3:** Phagocytic activity of crucian carp leukocytes.

Group	Phagocytic percentage (*PP* %)	Phagocytic index (*PI* %*)*
Control	36 ± 4.01	60 ± 4.20
MCR-EE	66.76 ± 9.63^**^	169.47 ± 20.37^**^

^**^ *p* < 0.01 (compared with control).

### 3.5 Immunoprotective rate of MCR-EE against bacteria

**Figure 4:**
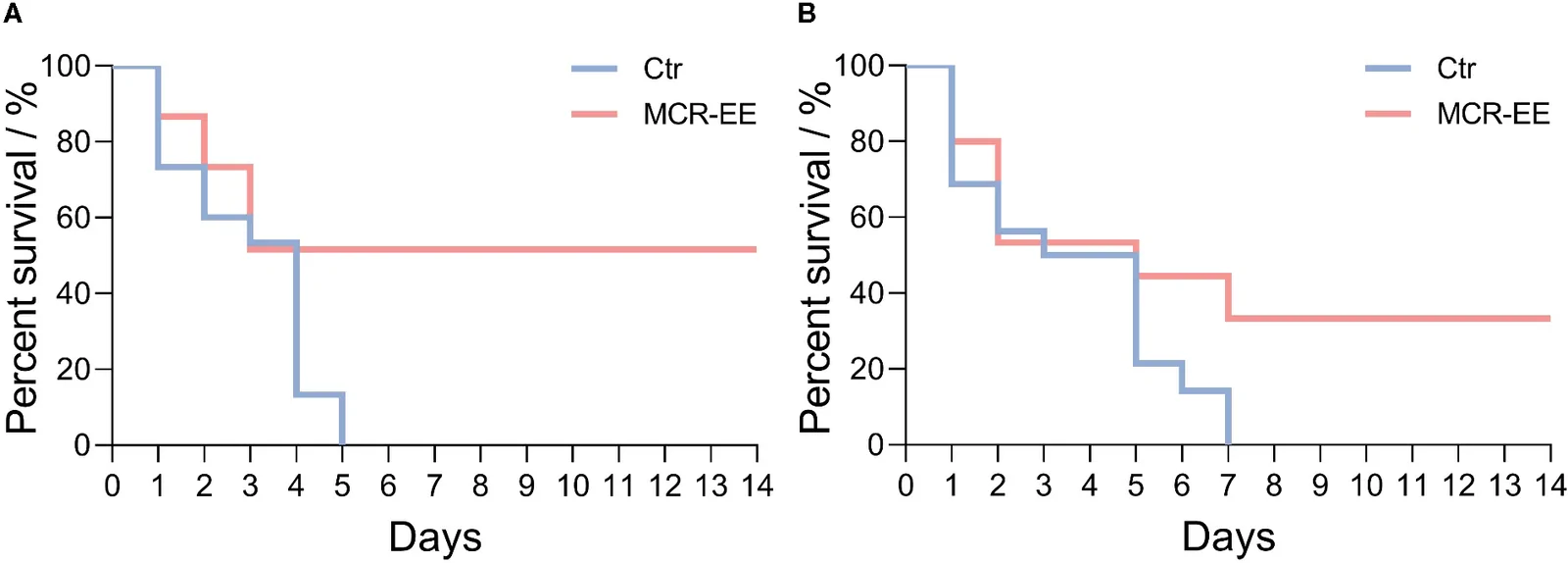
Survival rates of fish fed with MCR-EE and challenged with *A. veronii* **(A)** and *P. aeruginosa* **(B)**. Ctr indicates the control group.

Crucian carp were fed with MCR-EE and then challenged with *A. veronii* and *P. aeruginosa*. The results showed that after bacterial challenge, fish in both the control group and the MCR-EE group exhibited sluggish swimming and a sharp decrease in food intake. In the control group challenged with *A. veronii* and *P. aeruginosa*, all fish died within 5 days and 7 days, respectively. In the MCR-EE group, mortality stabilized after 7 days (**Figure 4**). The immunoprotective rates of MCR-EE against *A. veronii* and *P. aeruginosa* were 46.67% (*p* < 0.01) and 40% (*p* < 0.01), respectively (**Table 4**). These results indicate that immunization with MCR-EE enhances the ability of crucian carp to resist bacterial infection.

**Table 4:** Protective efficacy of MCR-EE in crucian carp challenged with *A. veronii* or *P. aeruginosa*.

Bacteria	Group	Total (no.)	Survived (no.)	Death (no.)	ADR (%)	RPS (%)	RPS (*p*-value)
*A. veronii*	Control	15	0	15	100	--	--
	MCR-EE	15	7	8	53.33	46.67^**^	0.006
*P. aeruginosa*	Control	15	0	15	100	--	--
	MCR-EE	15	6	9	60	40^**^	0.008

^**^ *p* < 0.01 (compared with control group). ADR indicates accumulating death rate.

### 3.6 Bacterial load in the kidneys of fish

**Figure 5:**
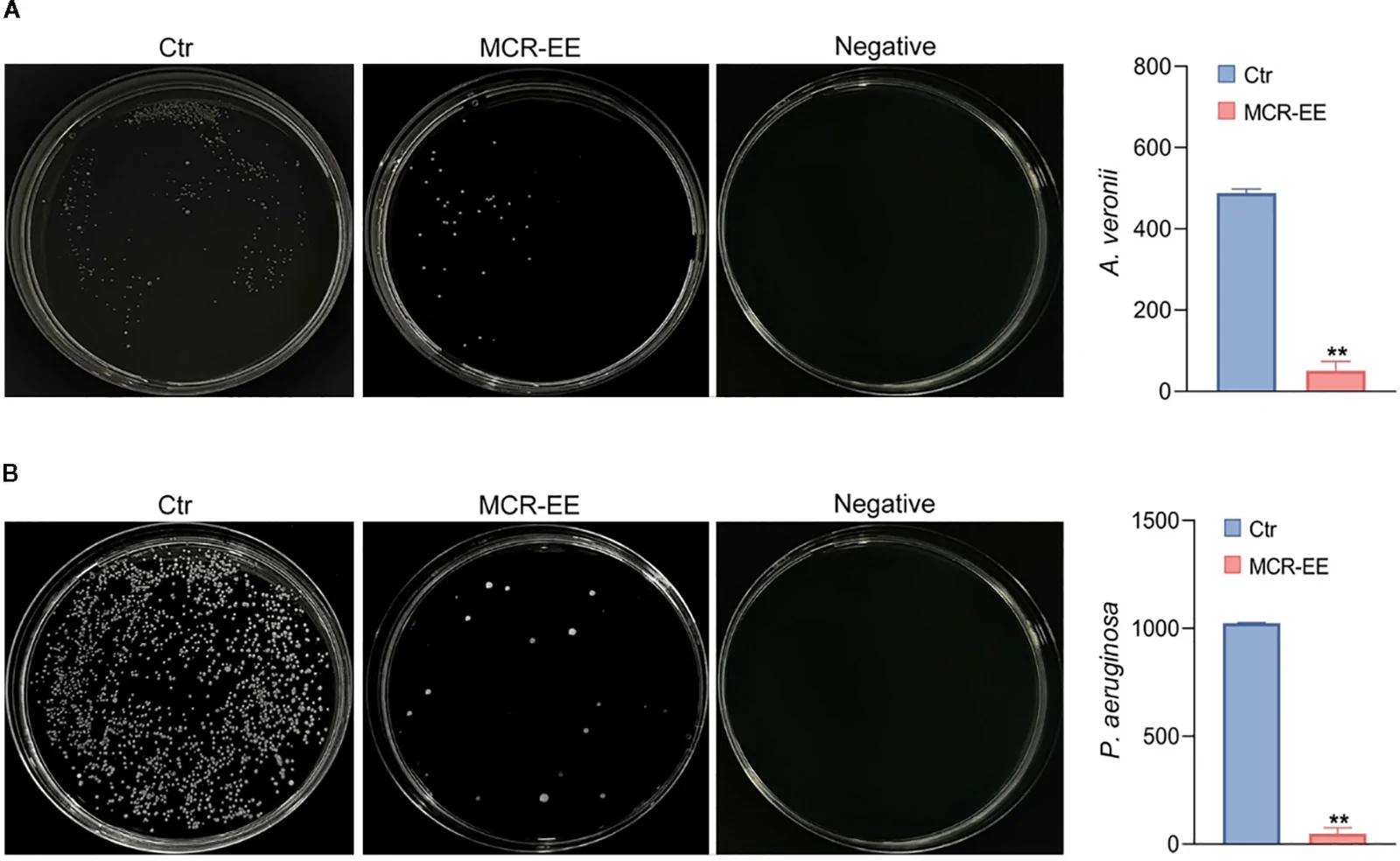
Spread plating on LB medium and colony counting assay of crucian carp kidneys. **(A, B)** represent the bacterial colonies and colony counts in culture plates after challenge with *A. veronii* and *P. aeruginosa*, respectively. Negative, indicates kidneys without bacterial challenging used as a negative control. ^**^p < 0.01 (compared to the control group).

Crucian carp were fed with MCR-EE and then challenged with *A. veronii* and *P. aeruginosa*. Kidney samples were collected from the crucian carp and subjected to spread plating on LB agar plates. The bacterial clearance effect of MCR-EE was evaluated by bacterial colony counting. The results showed that compared with the control group, the bacterial loads of *A. veronii* and *P. aeruginosa* in the kidneys of the MCR-EE group were significantly reduced (*p* < 0.01) (**Figure 5**).

### 3.7 Inflammatory factor mRNA expression

**Figure 6:**
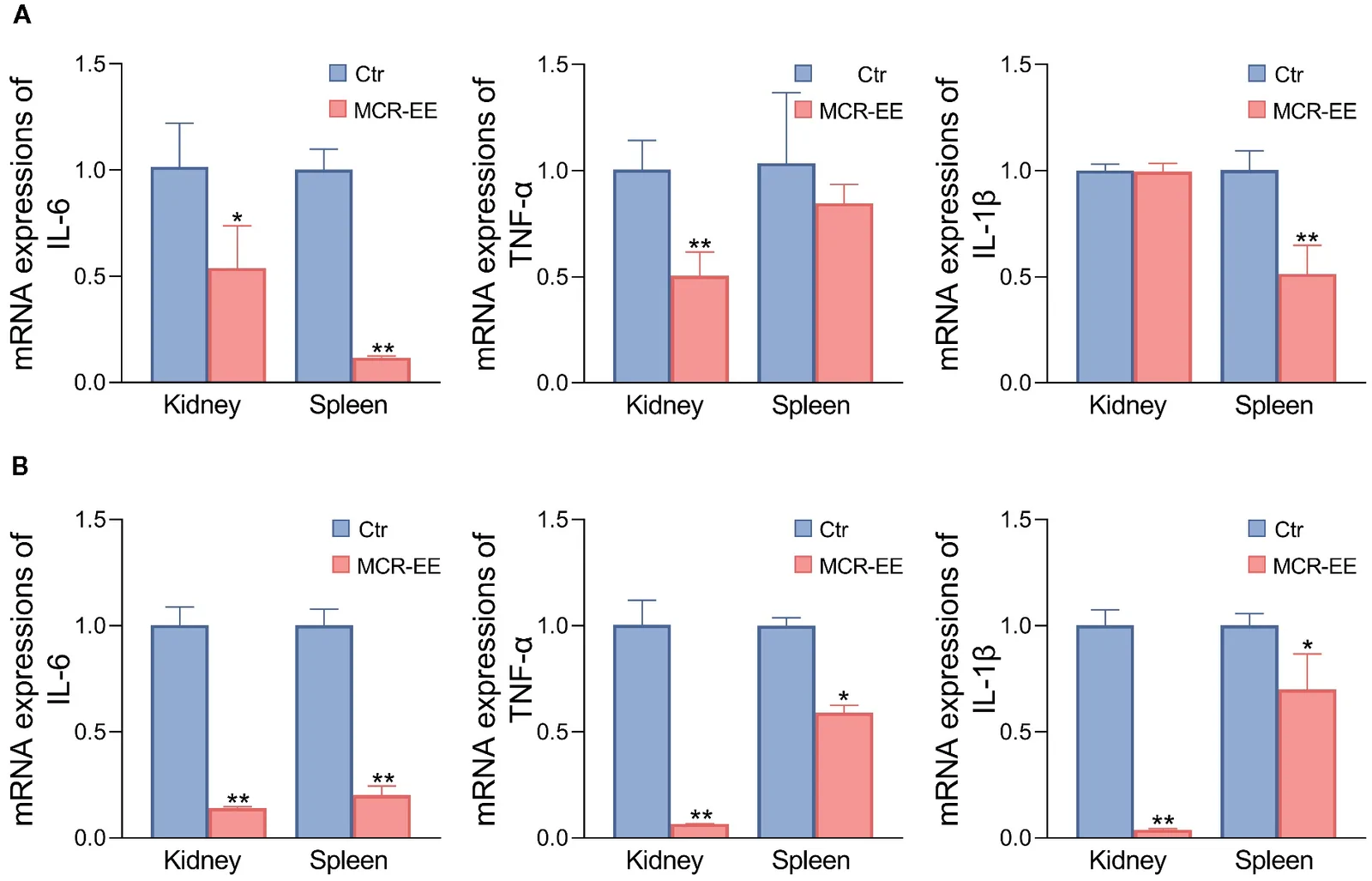
mRNA expression of inflammation-related factors in the kidney and spleen (n = 3 pooled samples). **(A, B)** represent challenge with *A. veronii* and *P. aeruginosa*, respectively. *p < 0.05, **p < 0.01 (compared to the control group).

Fish were fed with MCR-EE and challenged with *A. veronii* and *P. aeruginosa*. Two days later, total RNA was extracted from the kidney and spleen. The mRNA expression levels of the inflammatory factors of TNF-α, IL-6, and IL-1β were detected using qRT-PCR. The results are shown in **Figure 6**. The most mRNA expression levels of inflammatory factors in the MCR-EE group were decreased (*p* < 0.05). This indicates that immunization with MCR-EE reduces the inflammatory response induced by bacterial infection in fish, and that MCR-EE possesses anti-inflammatory activity.

### 3.8 mRNA expression of key pathway factors in antioxidant metabolism

**Figure 7:**
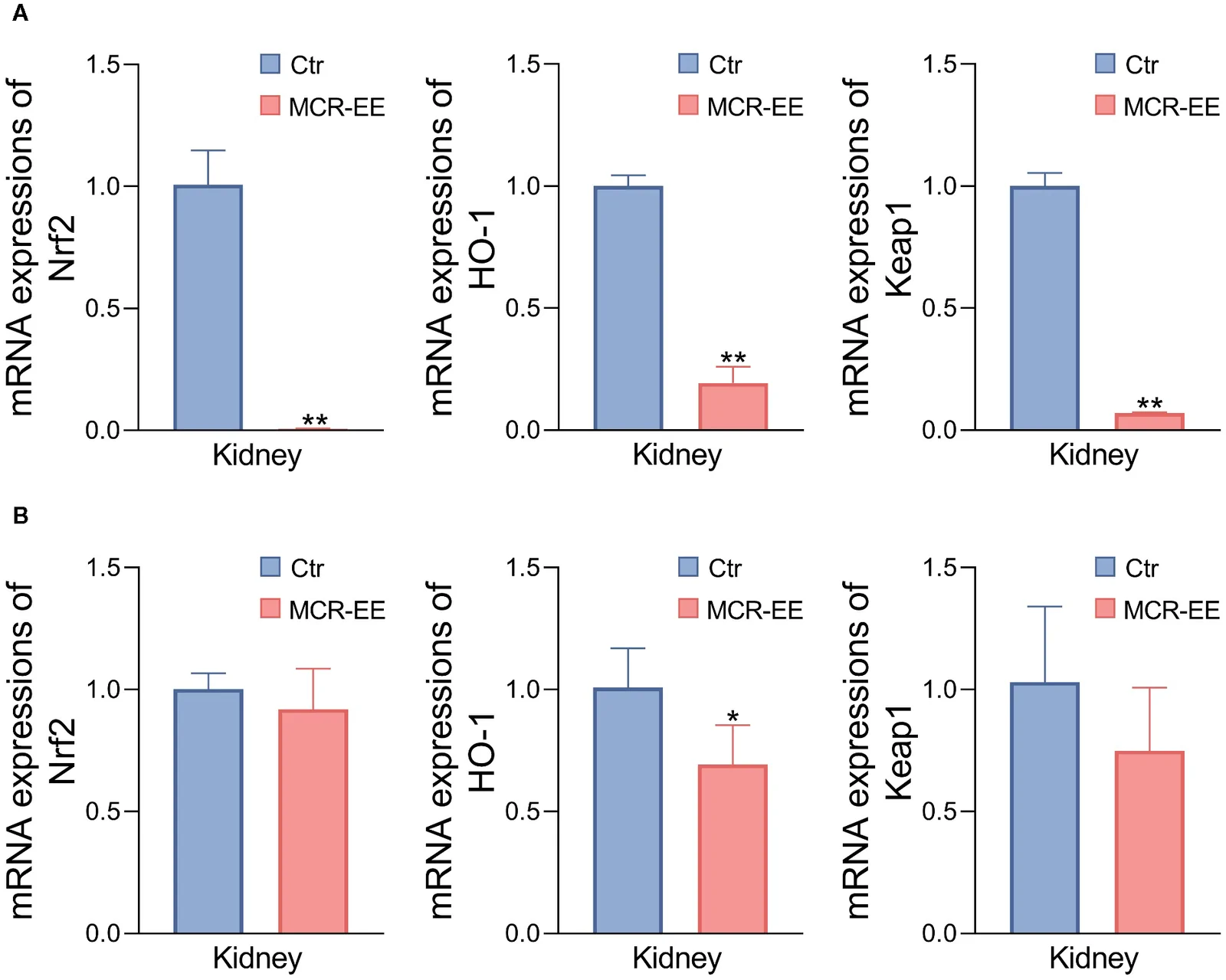
mRNA expression of key factors in the antioxidant metabolic pathway (n = 3 pooled samples). **(A, B)** represent challenge with *A. veronii* and *P. aeruginosa*, respectively. ^*^p < 0.05, ^**^p < 0.01 (compared to the control group).

Fish were continuously fed with MCR-EE for 7 days and then challenged with *A. veronii* and *P. aeruginosa*. Liver tissues were collected for qRT-PCR analysis. The results showed that the expression levels of most antioxidant pathway factor mRNAs (Nrf2, Keap1, HO-1) were decreased (*p* < 0.05) (**Figure 7**). This indicates that MCR-EE immunization reduced the oxidative stress response following bacterial infection in carp.

### 3.9 Antioxidant-related factors in the serum of crucian carp

**Figure 8:**
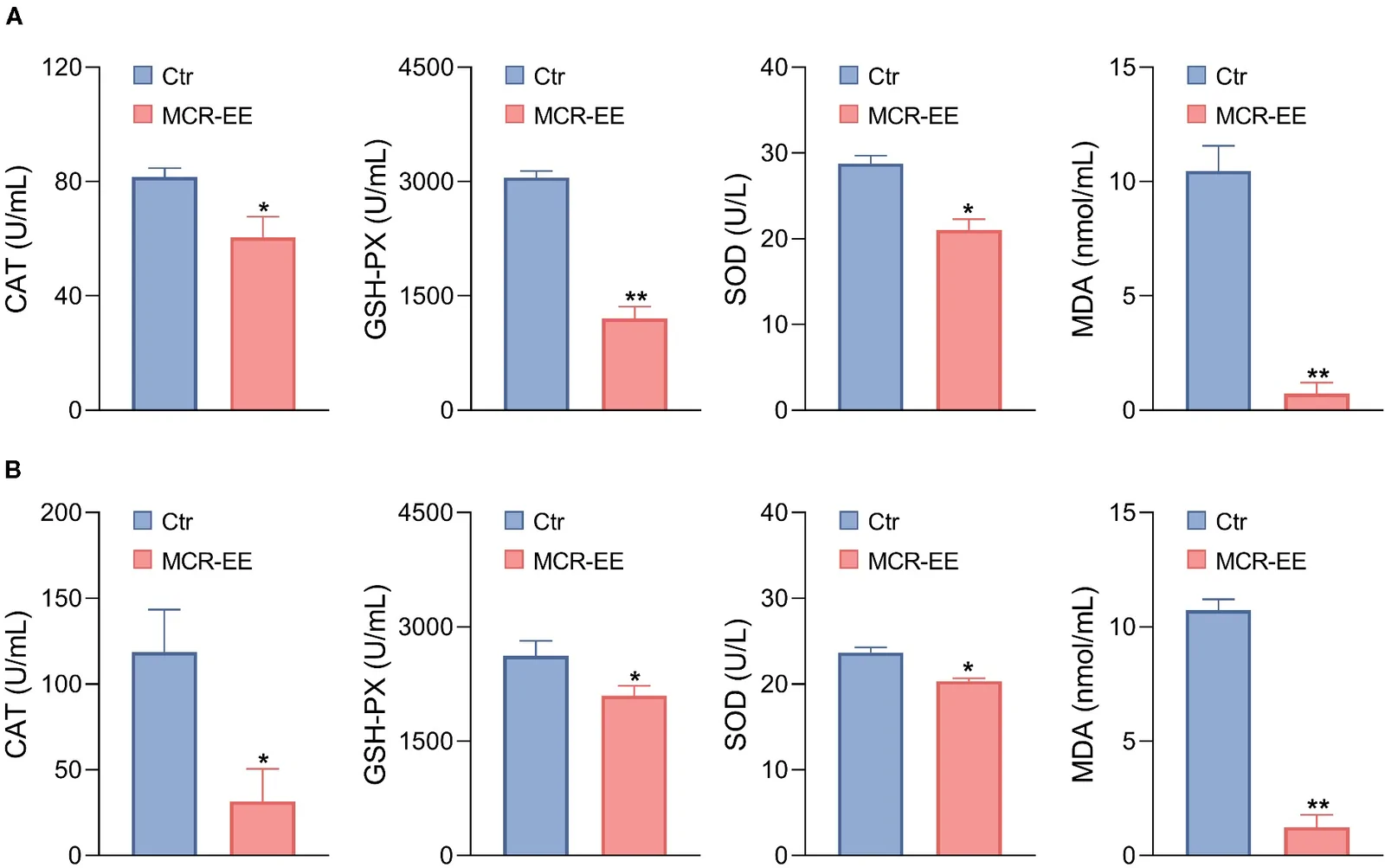
Analysis of antioxidant-related factors in the serum of crucian carp. **(A, B)** represent challenge with *A. veronii* and *P. aeruginosa*, respectively. ^*^p < 0.05, ^**^p < 0.01 (compared to the control group).

Crucian carp were continuously fed with MCR-EE for 7 days and then challenged with *A. veronii* and *P. aeruginosa*. Serum was collected from the fish for detection of antioxidant-related factors. The results showed that compared with the control group, the levels of antioxidant-related factors of GSH-Px, CAT, SOD, and MDA in the MCR-EE group were significantly decreased (*p* < 0.05) (**Figure 8**). This indicates that MCR-EE immunization reduced the oxidative stress response following bacterial infection in crucian carp.

### 3.10 Histopathological observation of crucian carp tissues

**Figure 9:**
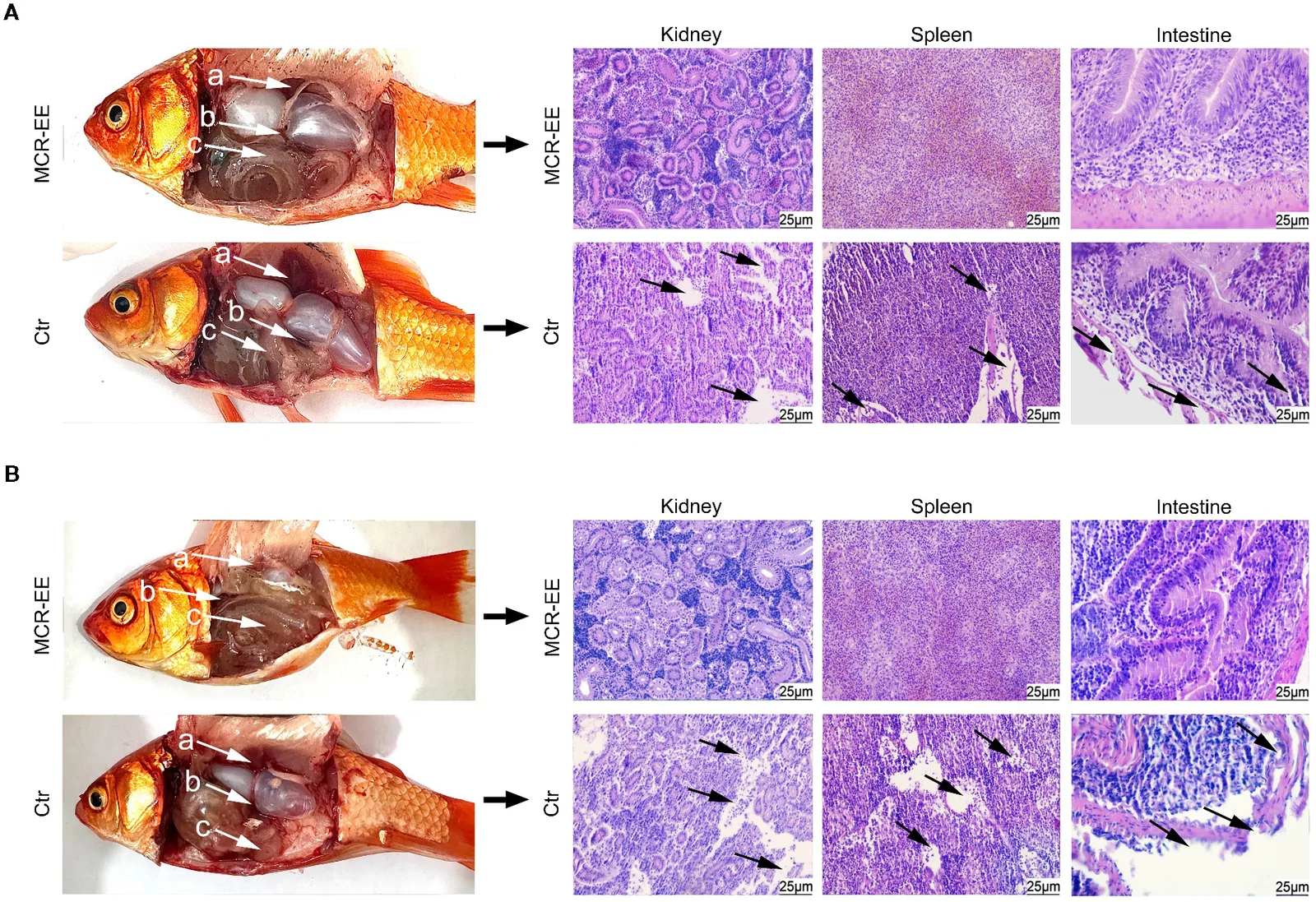
Histopathology of the kidney, spleen, and intestinal tissues of crucian carp. **(A, B)** represent challenge with *A. veronii* and *P. aeruginosa*, respectively.

Crucian carp were fed with MCR-EE for 7 days and then challenged with *A. veronii* and *P. aeruginosa*. Two days after bacterial challenge, the kidney, spleen, and intestine of the fish were collected for preparation of pathological sections. The protective effect of MCR-EE on the structural integrity of internal organs was analyzed through section observation. The results showed that in the control group, renal tubular and glomerular cells exhibited degeneration and apoptosis, with gaps present in the renal tissue structure; the spleen showed decreased nuclear density and loose, degenerated structure; the intestinal mucosa displayed loose cellular structure and cell apoptosis. In the MCR-EE group, the cellular structures of the kidney, spleen, and intestine remained intact, with no observable apoptosis or degeneration (**Figure 9**).

In addition, the histopathological damage scores of the kidney, spleen, and intestine in the MCR-EE group were lower than those in the control group (**Supplementary Figure 1**), indicating that dietary MCR-EE alleviated tissue damage in these organs.

### 3.11 Renal immunofluorescence analysis

**Figure 10:**
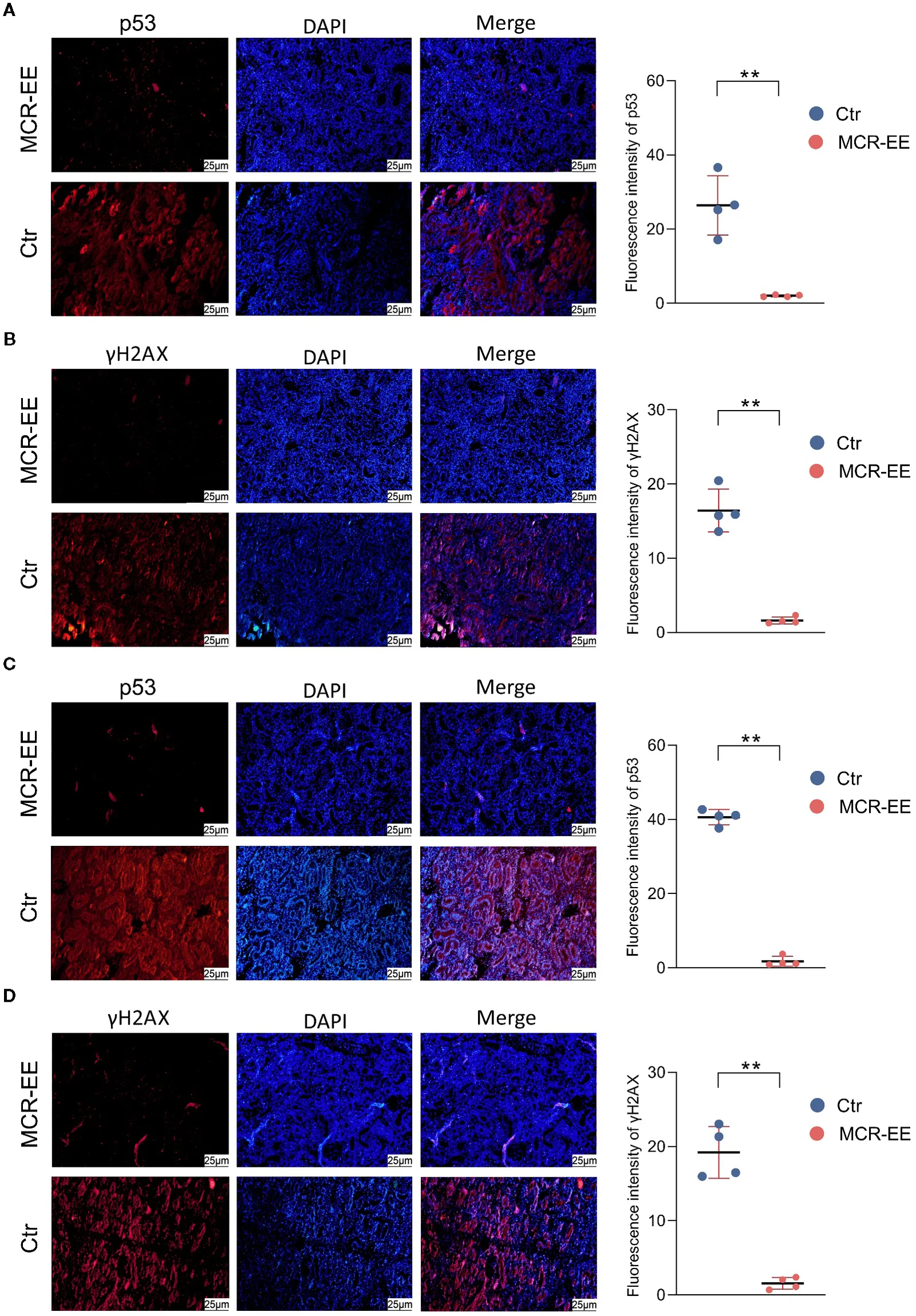
Immunofluorescence detection of kidney tissue. **(A, B)** represent challenge with *A. veronii*. **(C, D)** represent challenge with *P. aeruginosa*. **(A, C)** show the fluorescence intensity of p53, and **(B, D)** show the fluorescence intensity of γH2AX. ^**^p < 0.01 (compared with control).

Fish were fed with MCR-EE and then challenged with *A. veronii* and *P. aeruginosa*. Two days later, kidney tissues were collected from the fish to prepare tissue sections for immunofluorescence detection. The results are shown in **Figure 10**. Blue fluorescence indicates nuclear staining, while red fluorescence indicates p53 and γH2AX protein signals. The expression levels of these two proteins were analyzed based on fluorescence intensity. The results showed that the fluorescence intensities of p53 and γH2AX in the MCR-EE group were lower than those in the control group (*p* < 0.05) (**Figure 10**), indicating that the expression levels of p53 and γH2AX in kidney tissues were lower than those in the control group. Thus, these findings indirectly suggest MCR-EE can protect against renal cell apoptosis and DNA damage induced by bacterial infection.

## 4 Discussion

Plant alcohol extract refers to a mixture obtained from plant materials (roots, stems, leaves, flowers, fruits, etc.) through extraction and concentration methods such as maceration, reflux, or ultrasonic-assisted extraction using ethanol or methanol as solvents (36). Ethanol possesses good permeability and appropriate polarity, effectively dissolving flavonoids, alkaloids, terpenes, phenolic acids, glycosides, polysaccharides, proteins, and other components from plants (37). Alcohol extracts retain a variety of active ingredients from the original plant and are commonly used in bioactivity studies such as antioxidant, anti-inflammatory, antibacterial, and hypoglycemic research (38, 39). They are also important intermediates in the development of traditional Chinese medicine preparations, natural health products, and botanical pesticides. Jabbar et al. used 70% ethanol to extract active substances from *Vaccinium macrocarpon* L. and found that this alcohol extract exhibited antibacterial activity against uropathogenic bacteria (*Escherichia coli*, *Proteus vulgaris*, *Staphylococcus aureus*, *Enterococcus* sp., and coagulase-negative staphylococci) (31). Perić et al. discovered that an ethanol extract of South African geranium (*Pelargonium sidoides*) root has good immunomodulatory effects on acute rhinosinusitis (40). Zhang et al. used 80% ethanol to extract saponins from oats, and showed that the alcohol extract could serve as a natural anti-protein oxidant with application value in meat product processing and storage (41). In this study, MCR-EE was prepared using 70% ethanol. The contents of proteins, polysaccharides, and polyphenols of MCR-EE were found to be 0.70%, 0.44%, and 1.21%, respectively. The five small-molecule substances with the highest contents were L(+)-Arginine, 1-Deoxynojirimycin, 9,12,13-Todea, 4-Guanidinobutanoic acid, and Kelampayoside A in MCR-EE. The immunoprotective rates of MCR-EE immunization against *A. veronii* and *P. aeruginosa* were 46.67% and 40%, respectively. In preliminary experiments, we tested three administered doses of MCR-EE (100, 200, and 400 mg/kg body weight/day) in a small-scale fish trial (n = 10 per group) and found that the 200 mg/kg dose yielded the highest immunoprotection rate (60%) against *A. veronii* challenge (**Supplementary Table 3**). Therefore, the dietary dose of 200 mg/kg MCR-EE was selected for the evaluation of its immunomodulatory activity in this study. Furthermore, in the preliminary experiment, fish were fed MCR-EE at a dose of 200 mg/kg body weight/day. Compared with the control group (without MCR-EE supplementation), fish in the MCR-EE group exhibited normal feeding and swimming behaviors, with no mortality observed. Histological examination of visceral tissue sections revealed that the structural integrity of the kidney, spleen, and intestine in the MCR-EE group was consistent with that of the control group, with no pathological changes detected (**Supplementary Figure 2**). Thus, an MCR-EE alone group was not included in this study. In this study, the survival experiment and the other infection experiments used different bacterial doses. The lethal challenge (LD_50_) was used for the survival experiment to evaluate the protective efficacy of MCR-EE against pathogenic bacteria. The sublethal challenge (0.5 × LD_50_) was applied for all other post-infection analyses, including renal bacterial load, immune responses, oxidative stress, histopathology, and immunofluorescence assay. The lower dose of sublethal challenge ensured that all fish survived until the 2-day sampling point, thereby avoiding sample loss due to premature mortality. Collectively, the macromolecular and small-molecular substances in MCR-EE enhanced the resistance of crucian carp to aquatic bacteria.

Innate immunity serves as the body’s first line of defense against pathogen invasion. It does not require prior recognition of specific antigens, responds rapidly, and is a critical barrier for maintaining homeostasis and resisting infection. It can be evaluated through the activity of immune factors, the phagocytic activity of white blood cells, the attenuation of inflammatory responses, and the body’s ability to clear bacteria (42, 43). Xiao et al. immunized crucian carp with the outer membrane protein VF17320 of *Vibrio fluvialis* and found that the activities of immune factors (ACP, AKP, and LZM) were enhanced, the phagocytic capacity of white blood cells was increased, and the clearance of *Vibrio fluvialis* and *A. hydrophila* in fish was improved, indicating that VF17320 activated the innate immunity of fish (33). Li et al. found that the ethanol extract of *Abelmoschus manihot* reduced renal inflammatory responses and exhibited immunoenhancing effects (44). Mathew et al. extracted 80 Australian native medicinal plants with ethanol and discovered that 17 of them possessed anti-inflammatory activity and the ability to enhance innate immunity (45). In this study, after crucian carp were fed with MCR-EE, the activities of immune-related factors (ACP, AKP, and LZM) were significantly increased; the general phagocytic efficiency of white blood cells was enhanced; the mRNA expression of inflammatory cytokines in visceral tissues was downregulated, demonstrating the anti-inflammatory effects of MCR-EE, following bacterial infection (*A. veronii/P. aeruginosa*); and the kidney exhibited reduced bacterial loads. In this study, the elevated ACP, AKP, and LZM activities, together with enhanced phagocytosis, support immune-mediated bacterial clearance as the dominant protective mechanism. Nevertheless, the possibility of direct antibacterial effects of MCR-EE cannot be ruled out without additional experiments, such as fractionation and bioactivity-guided testing of individual components. Accordingly, future investigations should include systematic evaluations of the direct antibacterial activity of MCR-EE via MIC, MBC, growth-curve, and time-kill assays. Collectively, these findings indicate that MCR-EE immunization activates the innate immune response in crucian carp and improves their resistance against aquaculture bacterial pathogens.

MCR has been shown to reduce oxidative stress in mice by lowering the levels of inflammation-related factors such as MDA and scavenging ROS free radicals (46). It also exhibits significant antioxidant activity against lipid peroxidation in rat liver microsomes (47). Antioxidant activity reflects the ability of a substance to inhibit oxidative reactions, reduce oxidative damage, and scavenge free radicals, serving as a core indicator for evaluating whether a substance possesses antioxidant function. Typically, the expression of antioxidant factors is used to assess the oxidative stress level in animals (48, 49). Previous studies have shown that immunizing fish with IgY from live and inactivated *Vibrio fluvialis*, followed by challenge with different bacterial species, resulted in reduced expression of antioxidant factors, alleviated oxidative stress, and improved fish survival rates (50). Similarly, Chen et al. found that immunizing crucian carp with IgY antibodies from live or inactivated *A. veronii* reduced the oxidative stress level induced by bacterial infection (34). In this study, crucian carp were immunized with MCR-EE and then challenged with *A. veronii* and *P. aeruginosa*. The results showed significantly reduced the levels of antioxidant-related factors, as well as decreased mRNA levels of key factors in the antioxidant metabolic pathway (Nrf2, HO-1, and Keap1). When cells encounter oxidative stress, Keap1 acts as a key regulatory factor. The critical cysteine residues on the Keap1 protein are modified, leading to increased expression of Nrf2. Nrf2 translocates into the nucleus and binds to the antioxidant response element, initiating the transcription of cytoprotective target genes. Among these, HO-1 (heme oxygenase-1) is one of the most critical target genes. Once induced, HO-1 catalyzes the degradation of heme into carbon monoxide, free iron, and biliverdin, metabolites that possess antioxidant, anti-inflammatory, and cytoprotective functions. Therefore, the Keap1/Nrf2/HO-1 pathway constitutes a core defense mechanism against endogenous and exogenous oxidative damage in cells (51, 52). The observed reductions in mRNA levels of antioxidant factors and Keap1/Nrf2/HO-1 in fish, along with enhanced innate immunity, indicate that bacterial clearance is improved and the level of oxidative stress in the fish is reduced. In the qRT-PCR experiments for inflammation-related factors and antioxidant metabolic pathway factors, n = 3 pooled samples are the minimum acceptable sample size for statistical analysis and may limit the robustness of the gene-expression analysis. However, pooling is a common practice in qRT-PCR experiments to reduce individual variation, save costs, and meet the required RNA sample yield (33–35). In future studies, larger sample sizes (n ≥ 6) or individual-sample analyses would provide more robust the gene-expression analysis. Thus, MCR-EE attenuated the oxidative stress response induced by bacterial infection in fish.

The structural integrity of animal tissues and organs is a crucial guarantee for maintaining normal metabolic functions and serves as a key indicator for assessing the physiological effects and toxic damage of drugs (53). Histopathological section, characterized by its intuitiveness and accuracy, is an important technical method for observing microstructural morphology and diagnosing diseases (54). *Psychotria carthagenensis* is commonly used in religious rituals, and Nascimento et al. extracted its active components using ethanol and evaluated its toxicity by administering the ethanol extract to zebrafish, combined with histopathological observation of the liver and gills (55). Xiao et al. immunized crucian carp with the outer membrane protein VF08100 of *V. fluvialis* and subsequently challenged the fish with the pathogenic bacterium. They found that the histopathological structures of the intestine, kidney, and spleen remained intact, confirming that VF08100 protects the structural integrity of internal organs (56). In this study, crucian carp were fed with MCR-EE and then challenged with *A. veronii* and *P. aeruginosa*. The results showed that the structures of the intestine, kidney, and spleen remained intact, indicating that MCR-EE immunization preserved visceral structural integrity. Furthermore, using immunofluorescence technology, we observed reduced expression levels of p53 and γH2AX in kidney sections. The p53 protein is a core factor in the p53 signaling pathway, and its high expression can induce cell cycle arrest, DNA repair, cellular senescence, or apoptosis (57). γH2AX is the phosphorylated form of histone H2A.X at serine 139. Its core function is to recruit and anchor various DNA damage repair proteins to DNA break sites, and it is currently the most widely recognized marker of DNA double-strand breaks (58). Therefore, this study found that after MCR-EE immunization and bacterial challenge, the expression levels of p53 and γH2AX in the kidney were reduced, which indirectly suggests a decrease in apoptosis and DNA damage in the kidney. The further validation using TUNEL staining for apoptosis and the comet assay for DNA damage is required to confirm these findings. Collectively, MCR-EE immunization maintains the structural and physiological integrity of internal organs in fish following bacterial infection.

## 5 Conclusions

In summary, the MCR-EE was prepared, and the contents of both biological macromolecules and small molecules in MCR-EE were determined. Feeding MCR-EE to crucian carp was found to enhance the activity of immune factors and increase general leukocyte phagocytosis. Using a model in which crucian carp were fed with the MCR-EE and subsequently challenged with *A. veronii* and *P. aeruginosa*, it was found that MCR-EE increased the survival rate of fish, reduced visceral bacterial load, exerted effects in reducing inflammatory responses and the oxidative stress pathway, maintained the structural integrity of internal organs, and indirectly suggested a reduction in apoptosis and DNA damage in visceral cells. Therefore, given the abundant resources and low cost of MCR, MCR-EE has the potential to serve as a immunostimulant for the prevention and control of bacterial infections (*A. veronii* and *P. aeruginosa*) in fish.

## Data Availability

The original contributions presented in the study are included in the article/**Supplementary Material**. Further inquiries can be directed to the corresponding authors.

## References

[1] Elsler LG Gephart JA Zamborain-Mason J Cashion T Troell M Naylor RL . Global nutritional equity of fishmeal and aquaculture trade flows. Proc Natl Acad Sci USA. (2026) 123:e2506699123. doi: 10.1073/pnas.2506699123 41662524 PMC12912983

[2] Rojas-Peña M Aceituno P Ordóñez-Grande B García-Ordoñez M Liang X Okeleye O . Oral antiviral vaccines in aquaculture: Current status, challenges, and future prospects. Fish Shellfish Immunol. (2026) 168:110962. doi: 10.1016/j.fsi.2025.110962 41138895

[3] Vu NT Phuc TH Huong TTM Nguyen NH . First high-density linkage map and quantitative trait loci for disease resistance in striped catfish Pangasianodon hypophthalmus. Int J Mol Sci. (2026) 27:784. doi: 10.3390/ijms27020784 41596438 PMC12841340

[4] Bai Y Li X Yan W Zhao L Huang L Yan Q . The detection of Edwardsiella tarda by aptamer-based qPCR. Front Vet Sci. (2025) 12:1635525. doi: 10.3389/fvets.2025.1635525 40771951 PMC12327390

[5] Gao KP Dou JR Ma BB Ma TB Li N Qian AD . Engineered Lactobacillus casei targets the IgT-pIgR axis to confer mucosal protection against Aeromonas veronii in snakehead (Channa argus). Front Immunol. (2026) 17:1759765. doi: 10.3389/fimmu.2026.1759765 41948342 PMC13050950

[6] Rimphanitchayakit V Supungul P Songserm T Phuttikul J Tassanakajon A Visetnan S . Functional characterization of PmPen5 from the black tiger shrimp Penaeus monodon. Fish Shellfish Immunol. (2026) 174:111385. doi: 10.1016/j.fsi.2026.111385 42055177

[7] Uno Y Umeki K Yamada A Hashikura Y Nomura H Hayashi M . Development of polymerase chain reaction assays specific for individual pathogenic Aeromonas species. J Microbiol Methods. (2025) 235:107142. doi: 10.1016/j.mimet.2025.107142 40324564

[8] Lin Y Zhang Q Chen L Liu Y Lin X Peng X . Neomycin affects cardiovascular and hematopoietic system via the PI3K/Akt pathway in zebrafish larvae. Ecotoxicol Environ Saf. (2025) 296:118203. doi: 10.1016/j.ecoenv.2025.118203 40262245

[9] Wang J Qin T Chen K Pan L Xie J Xi B . Antimicrobial and antivirulence activities of carvacrol against pathogenic Aeromonas hydrophila. Microorganisms. (2022) 10:2170. doi: 10.3390/microorganisms10112170 36363761 PMC9699308

[10] Ofosu-Koranteng F Affordofe M Botwe PK Tettey P Duodu CP Udofia EA . Levels of tetracycline and sulphonamide residues in freshwater fish: a systematic review and meta-analysis. Sci Total Environ. (2026) 1023:181605. doi: 10.1016/j.scitotenv.2026.181605 41785574

[11] Zhang J Ip JC Hamed M Lee JS Mo J . A review on antibiotic use in tilapia farming: Pharmacokinetics, impacts, and potential health risks. Mar pollut Bull. (2026) 228:119582. doi: 10.1016/j.marpolbul.2026.119582 41855966

[12] Zhao X Lin S Wang J Wu S Ren C Sun X . Nelumbinis Plumula attenuates high-fat-induced inflammation in non-alcoholic fatty liver through the NOX/NF-κB pathway. J Ethnopharmacol. (2026) 355:120663. doi: 10.1016/j.jep.2025.120663 41052689

[13] Park SH . Recent research on the role of phytochemicals from ginseng in management of osteosarcoma, osteoporosis, and osteoarthritis. Nutrients. (2025) 17:1910. doi: 10.3390/nu17111910 40507179 PMC12158031

[14] Tao X Li Y Liu S Wu W Wu J Men X . Development of multi-bioactive driven composite plant extracts and functional study in mice and piglets. Antioxidants. (2026) 15:468. doi: 10.3390/antiox15040468 42072110 PMC13114034

[15] Shuid AN Mohd Nawi SFA Kamarudin SN Shuid AN Abdul Malik MM Abu Hanipah NF . Exploring the antibacterial properties of acmella species: a systematic literature review. Drug Des Devel Ther. (2026) 20:536279. doi: 10.2147/DDDT.S536279 41836524 PMC12983169

[16] Silva B Hernández V Carrasco C Fuica C Contreras F Deride M . Antibacterial and biostimulatory activity of urtica magellanica extract on fish pathogenic bacteria and CHSE-214 salmonid cell line. Nat Prod Res. (2026) 17:1–7. doi: 10.1080/14786419.2026.2658148 41995060

[17] Shin SM Mendis WRH Kang SY Jeong HJ Kim T Jung SJ . Efficacy of a combined natural extract of turmeric (Curcuma longa) and liquorice (Glycyrrhiza uralensis) against viral haemorrhagic septicaemia virus in olive flounders (Paralichthys olivaceus). J Fish Dis. (2026) 49:e70002. doi: 10.1111/jfd.70002 40576163 PMC12667007

[18] Fazio A Cerezuela R Panuccio MR Cuesta A Esteban MÁ . *In vitro* effects of Italian Lavandula multifida L. leaf extracts on gilthead seabream (Sparus aurata) leucocytes and SAF-1 cells. Fish Shellfish Immunol. (2017) 66:334–44. doi: 10.1016/j.fsi.2017.05.033 28522420

[19] Zhai Q Chang Z Li J Li J . Protective effects of combined utilization of quercetin and florfenicol on acute hepatopancreatic necrosis syndrome infected Litopenaeus vannamei. Antibiotics. (2022) 11:1784. doi: 10.3390/antibiotics11121784 36551441 PMC9774288

[20] Giri SS Jun JW Lee SB Joo SJ Hwang MH Park JH . Effect of papaya leaf extract on growth performance, skin mucosal immune responses, liver enzymes, disease resistance, and tight junctions of Cyprinuscarpio. Fish Shellfish Immunol. (2026) 168:110916. doi: 10.1016/j.fsi.2025.110916 41077094

[21] Guo Z Wang L Zhou J Tong Y Zhang J Guan M . The effects of the sanhuanglianqiao mixture on Vibrio parahaemolyticus infection in Penaeus vannamei. Microb Pathog. (2025) 206:107735. doi: 10.1016/j.micpath.2025.107735 40412734

[22] Jara-Medina NR Cueva DF Cedeño-Pinargote AC Gualle A Aguilera-Pesantes D Méndez MÁ . Eco-alternative treatments for Vibrio parahaemolyticus and V. cholerae biofilms from shrimp industry through Eucalyptus (Eucalyptus globulus) and Guava (Psidium guajava) extracts: a road for an Ecuadorian sustainable economy. PloS One. (2024) 19:e0304126. doi: 10.1371/journal.pone.0304126 39137207 PMC11321589

[23] Zeng XX Gao DD Zhang F . Cortex Mori Radicis [Morus Alba L. (Moraceae)] extract alleviates senescence via PI3K/Akt signaling in COPD fibroblasts. Phytomedicine. (2024) 135:156004. doi: 10.1016/j.phymed.2024.156004 39326135

[24] Lyu YR Yang WK Lee SW Kim SH Kim DS Son E . Inhibitory effects of modified gamgil-tang in a particulate matter-induced lung injury mouse model. J Ethnopharmacol. (2022) 284:114789. doi: 10.1016/j.jep.2021.114789 34728315

[25] Yue X Han J Pan C Zhang Y Liu S Gao F . Xiebai san alleviates allergic pulmonary inflammation by modulating arachidonic acid metabolism. Pharmaceuticals. (2026) 19:440. doi: 10.3390/ph19030440 41901287 PMC13029534

[26] Chen X Han Y Chen L Tian QL Yin YL Zhou Q . Discovery and characterization of the flavonoids in Cortex Mori Radicis as naturally occurring inhibitors against intestinal nitroreductases. Chem Biol Interact. (2022) 368:110222. doi: 10.1016/j.cbi.2022.110222 36244406

[27] Cui X Zhu XC Yao SQ Wang R Zhang YX Li J . Phytochemical profiling of mulberry diels-alder adducts as selective butyrylcholinesterase inhibitors: *in vitro* activity, molecular docking, and molecular dynamics simulation. Molecules. (2026) 31:1574. doi: 10.3390/molecules31101574 42197128 PMC13209706

[28] Han S Kim H Lee MY Lee J Ahn KS Ha IJ . Anti-cancer effects of a new herbal medicine PSY by inhibiting the STAT3 signaling pathway in colorectal cancer cells and its phytochemical analysis. Int J Mol Sci. (2022) 23:14826. doi: 10.3390/ijms232314826 36499154 PMC9740770

[29] Hong S Cho HR Kim JH Kim M Lee S Yang K . Suppression of bone resorption by Mori Radicis Cortex through NFATc1 and c-Fos signaling-mediated inhibition of osteoclast differentiation. J Chin Med Assoc. (2024) 87:615–26. doi: 10.1097/JCMA.0000000000001096 38651853 PMC12718918

[30] Kim DS Lee HY Kim HJ Lee GH Lim YJ Ko BM . Combined treatment of Mori folium and Mori Cortex Radicis ameliorate obesity in mice via UCP-1 in brown adipocytes. Nutrients. (2023) 15:3713. doi: 10.3390/nu15173713 37686745 PMC10489681

[31] Jabbar Al Kaabi HK Hmood BA . Antimicrobial activity of cranberry juice (Vaccinium macrocarpon L.) ethanol extract against uropathogenic bacteria. Open Vet J. (2025) 15:813–9. doi: 10.5455/OVJ.2025.v15.i2.30 40201815 PMC11974282

[32] Yang C Cui C Zhu Y Xia X Jin G Liu C . Effect of brewing conditions on the chemical and sensory profiles of milk tea. Food Chem X. (2022) 16:100453. doi: 10.1016/j.fochx.2022.100453 36185102 PMC9516450

[33] Xiao H Cui P Chen J Meng L Che X Ma Z . Evaluation of the multivalent immune protective effects of the Vibrio fluvialis outer membrane protein VF17320, and its DNA and IgY antibody vaccines in fish. Front Vet Sci. (2025) 12:1586258. doi: 10.3389/fvets.2025.1586258 40607340 PMC12213336

[34] Chen J Cui P Xiao H Wu X Lu J Liu Y . The passive immunoprotective activity using egg yolk IgY antibodies of live or inactivated Aeromonas veronii against major pathogenic bacteria (A. veronii and A. hydrophila) in fish. Vet Sci. (2025) 12:831. doi: 10.3390/vetsci12090831 41012756 PMC12474119

[35] Cui P Chen J Ma Z Xiao H Lu J Wang J . The multivalent passive immune-protective vaccines of egg yolk immunoglobulin Y derived from live or inactivated Pseudomonas anguilliseptica against aquaculture pathogens (P. anguilliseptica and Aeromonas veronii) in goldfish (Carassius auratus). Front Mar Sci. (2025) 12:1655485. doi: 10.3389/fmars.2025.1655485

[36] Govindhan P . Phytochemical analysis of the methanolic extract from the mangrove species Avicennia marina plant species inhabited in coastal water. Nat Prod Res. (2026) 40:215–25. doi: 10.1080/14786419.2024.2402474 39267342

[37] Rezaei F Eikani MH Nosratinia F Bidaroni HH . Optimization of ethanol-modified subcritical water extraction of curcuminoids from turmeric (Curcuma longa L.) rhizomes: Comparison with conventional techniques. Food Chem. (2023) 410:135331. doi: 10.1016/j.foodchem.2022.135331 36610095

[38] Fujii T Yazawa A Isobe T Kitada K Takai Y Yu Y . Analysis of the proliferation-inhibiting active components against periodontal pathogenic bacteria contained in fig leaf extract. FEMS Microbiol Lett. (2026) 373:fnaf147. doi: 10.1093/femsle/fnaf147 41489468

[39] Zhang X Cai J Chen L Tai J Liu J Cao Y . Gastrodin ameliorates DSS-induced ulcerative colitis via enhancing the abundance of Akkermansia muciniphila and modulating the metabolites. Phytomedicine. (2025) 146:157138. doi: 10.1016/j.phymed.2025.157138 40779823

[40] Perić A Vezmar Kovačević S Barać A Perić A Vojvodić D . Immunomodulatory effects of Pelargonium sidoides extract (EPs7630) in the treatment of acute rhinosinusitis. Expert Rev Mol Med. (2025) 27:e25. doi: 10.1017/erm.2025.10013 40660733 PMC12315653

[41] Zhang L Li J Huo Y Yang W Chen J Gao Z . Ultrasonic extraction and antioxidant evaluation of oat saponins. Ultrason Sonochem. (2024) 109:106989. doi: 10.1016/j.ultsonch.2024.106989 39059252 PMC11327440

[42] Ding Z Wang X Liu Y Zheng Y Li H Zhang M . Dietary mannan oligosaccharides enhance the non-specific immunity, intestinal health, and resistance capacity of juvenile blunt snout bream (Megalobrama amblycephala) against Aeromonas hydrophila. Front Immunol. (2022) 13:863657. doi: 10.3389/fimmu.2022.863657 35784342 PMC9240629

[43] Tian X Liu J Li Y Wang Y Luo Y He H . Agonists for cytosolic bacterial receptor ALPK1 induce antitumour immunity. Nature. (2026) 650:242–50. doi: 10.1038/s41586-025-09828-9 41372408

[44] Li N Tang H Wu L Ge H Wang Y Yu H . Chemical constituents, clinical efficacy and molecular mechanisms of the ethanol extract of Abelmoschus manihot flowers in treatment of kidney diseases. Phytother Res. (2021) 35:198–206. doi: 10.1002/ptr.6818 32716080 PMC7891592

[45] Mathew S Zhou X Münch G Raju R . Exploring the anti-inflammatory potential of Australian native plants based on their ethnopharmacological knowledge. Chem Biodivers. (2024) 21:e202400492. doi: 10.1002/cbdv.202400492 38700281

[46] You S Kim GH . Protective effect of Mori Cortex radicis extract against high glucose-induced oxidative stress in PC12 cells. Biosci Biotechnol Biochem. (2019) 83:1893–900. doi: 10.1080/09168451.2019.1621154 31130105

[47] Lu AQ Chen MH Gao J Wang L Yang HY Li L . A Tri-O-bridged diels-alder adduct from Cortex Mori Radicis. Molecules. (2018) 23:133. doi: 10.3390/molecules23010133 29315271 PMC6017575

[48] Huang H Wang L Shi F Liu J Li H Zuo J . The protective effect and mechanism of phycocyanin on diabetic retinopathy in rats. Tissue Cell. (2026) 99:103228. doi: 10.1016/j.tice.2025.103228 41260006

[49] Boutabet K Harrouche K Kebsa W Bounar H Ceylan FD Capanoglu E . Antioxidant and gastroprotective effect of Erica arborea extract against diclofenac-induced gastric ulcer in mice: *in vivo* and in silico molecular docking study. Chem Biodivers. (2026) 23:e01416. doi: 10.1002/cbdv.202501416 41237065 PMC13420696

[50] Liu X Xiao H Cui P Chen J Chao J Wu X . Differential polyvalent passive immune protection of egg yolk antibodies (IgY) against live and inactivated Vibrio fluvialis in fish. Fish Shellfish Immunol. (2024) 51:109751. doi: 10.1016/j.fsi.2024.109751 38971349

[51] Luo L Huang F Zhong S Ding R Su J Li X . Astaxanthin attenuates ferroptosis via Keap1-Nrf2/HO-1 signaling pathways in LPS-induced acute lung injury. Life Sci. (2022) 311:121091. doi: 10.1016/j.lfs.2022.121091 36252699

[52] Mao LW Jiang QY Meng N Xiao L Zhang Q Chen YX . Sirt6 promotes DNA damage repair in osteoarthritis chondrocytes by activating the Keap1/Nrf2/HO-1 signaling pathway. Cell Cycle. (2024) 23:205–17. doi: 10.1080/15384101.2024.2316493 38389322 PMC11037281

[53] Wei HK Yu CD Hu B Zeng X Ichise H Li L . Tumour-brain crosstalk restrains cancer immunity via a sensory-sympathetic axis. Nature. (2026) 650:1007–16. doi: 10.1038/s41586-025-10028-8 41639447 PMC12935554

[54] Mironova YA Dang B Heo D Xu YKT Hsu AY Eugenin von Bernhardi J . Myelin is repaired by constitutive differentiation of oligodendrocyte progenitors. Science. (2026) 391:eadu2896. doi: 10.1126/science.adu2896 41570153 PMC12997438

[55] Nascimento GCZ Matias R Miranda-Vilela AL Farias KS Silva DB Facco GG . Acute exposure of zebrafish (Danio rerio) adults to psychotria carthagenensis leaf extracts: chemical profile, lack of genotoxicity and histological changes. Drug Chem Toxicol. (2024) 47:1358–68. doi: 10.1080/01480545.2024.2367560 38953234

[56] Xiao H Duan S Cui P Chen J Che X Lu J . Polyvalent immunoprotection of protein, DNA and IgY antibody vaccines of Vibrio fluvialis outer membrane protein VF08100 in fish. Fish Shellfish Immunol. (2025) 161:110260. doi: 10.1016/j.fsi.2025.110260 40064214

[57] Stanage TH Li S Segura-Bayona S Idilli AI Millar R Hewitt G . RPA exhaustion activates SLFN11 to eliminate cells with heightened replication stress. Nat Cell Biol. (2026) 28:240–54. doi: 10.1038/s41556-025-01852-1 41514018 PMC12904793

[58] Radhakrishnan M Parida BP Shekhar H Singh P Ansari MA Singh J . Anticancer potential of Curcuma caesia: induction of DNA damage and apoptosis in cervical cancer. BMC Complement Med Ther. (2026) 26:160. doi: 10.1186/s12906-026-05351-0 41862889 PMC13127080

