# Supplementary material for: Identification of the immunoprotective activity of Mori Cortex Radicis ethanol extract against aquaculture pathogens (Aeromonas veronii, Pseudomonas aeruginosa)
Source: Front Immunol. 2026 Aug 17;17:1927434. doi: 10.3389/fimmu.2026.1927434 (PMC13526930; doi:10.3389/fimmu.2026.1927434)
Supplement: Supplementary file 1 [file DataSheet1.zip › Supplementary files/Supplementary Figure S1.docx]

A

B

**Supplementary Figure S1** Histopathological damage quantitative score of kidney, spleen and intestine. A and B represent challenge with *A. veronii* and *P. aeruginosa*, respectively. Data are presented as the mean ± SD (n = 3). ^*^*p* < 0.05, ^**^*p* < 0.01 (compared with control).
