# Supplementary material for: Identification of the immunoprotective activity of Mori Cortex Radicis ethanol extract against aquaculture pathogens (Aeromonas veronii, Pseudomonas aeruginosa)
Source: Front Immunol. 2026 Aug 17;17:1927434. doi: 10.3389/fimmu.2026.1927434 (PMC13526930; doi:10.3389/fimmu.2026.1927434)
Supplement: Supplementary file 1 [file DataSheet1.zip › Supplementary files/Supplementary Figure S2.docx]

**Supplementary Figure S2** Effects of dietary MCR-EE on the visceral tissue structure of fish.
