# Supplementary material for: Identification of the immunoprotective activity of Mori Cortex Radicis ethanol extract against aquaculture pathogens (Aeromonas veronii, Pseudomonas aeruginosa)
Source: Front Immunol. 2026 Aug 17;17:1927434. doi: 10.3389/fimmu.2026.1927434 (PMC13526930; doi:10.3389/fimmu.2026.1927434)
Supplement: Supplementary file 1 [file DataSheet1.zip › Supplementary files/Supplementary Table S1.docx]

**Supplement Table S1** qRT-PCR primers for the evaluation of inflammation-related genes.

| **Proteins** | **NCBI**  **accession number** | **Forward primer (5’-3’)** | **Reverse primer (5’-3’)** |
| --- | --- | --- | --- |
| GAPDH | XM_026284269.1 | GATTTCAACGGGGATGTGCG | TCACACACACGGTTGCTGTA |
| IL-6 | XM_026289280.1 | TCTCCTCAGACCCTCAGACG | CGTTTGGTCCCGTGTTTGAC |
| IL-8 | XM_026267284.1 | GGAGTGCAGGCCACTGTTAG | ATCAGAAGCATGAAGGCGGA |
| TNF-α | EU069817.1 | GGGCCACATCGTGATTCGTA | GCCTCCAGTGTAGCATGTGT |
| IL-1β | AJ249136.1 | TTCAGGAAAGAGACGGGCAC | GTCAGTTGGCACCTGGATCA |
| Nrf2 | XM_042763189.1 | ATCTGCACAGCTTCTCACCT | AGTCAGAGTCGCTGAAACCA |
| HO-1 | KC758864.1 | CAGCTCTCCAGTGCTACAGT | TCAGCCTGTGGATTTGACCT |
| Keap1 | XP_026101140.1 | ATGTACCAGATCGACAGCGT | TTGAAGAACTCCTCCTGCGT |
