# Supplementary material for: Identification of the immunoprotective activity of Mori Cortex Radicis ethanol extract against aquaculture pathogens (Aeromonas veronii, Pseudomonas aeruginosa)
Source: Front Immunol. 2026 Aug 17;17:1927434. doi: 10.3389/fimmu.2026.1927434 (PMC13526930; doi:10.3389/fimmu.2026.1927434)
Supplement: Supplementary file 1 [file DataSheet1.zip › Supplementary files/Supplementary Table S2.docx]

**Supplementary Table S2** LC-MS analysis of small molecule compounds in MCR-EE.

| NO. | Metabolite | Formula | M/Z | Retention time | Fragmentation Score | Mode | Abundance |
| --- | --- | --- | --- | --- | --- | --- | --- |
| 1 | L(+)-Arginine | C6H14N4O2 | 175.1188 | 0.5607 | 93.2 | pos | 21031937 |
| 2 | 1-Deoxynojirimycin | C6H13NO4 | 164.0916 | 0.5252 | 96 | pos | 9844135.342 |
| 3 | 9,12,13-Todea | C18H34O5 | 329.233 | 8.3127 | 91.3 | neg | 9774943.176 |
| 4 | 4-Guanidinobutanoic acid | C5H11N3O2 | 146.0922 | 0.6321 | 94.7 | pos | 6862460.962 |
| 5 | Kelampayoside A | C20H30O13 | 477.1608 | 3.1636 | 79.5 | neg | 6511341.679 |
| 6 | Gorlic acid | C18H30O2 | 279.2314 | 13.0367 | 92.9 | pos | 6445849.67 |
| 7 | Citric acid | C6H8O7 | 191.0189 | 0.8365 | 97.6 | neg | 6143575.612 |
| 8 | 9(s)-hode | C18H32O3 | 295.2274 | 13.055 | 95.1 | neg | 5686704.173 |
| 9 | Octadec-9-ene-1,18-dioic-acid | C18H32O4 | 311.2225 | 12.411 | 86.8 | neg | 4849697.651 |
| 10 | Chlorogenic Acid | C16H18O9 | 353.0875 | 2.8973 | 97.1 | neg | 4730333.365 |
| 11 | 9(S)-HOTrE | C18H30O3 | 295.2263 | 12.2675 | 90.8 | pos | 4567734.245 |
| 12 | 13-OxoODE | C18H30O3 | 295.2262 | 13.1696 | 96 | pos | 4248978.916 |
| 13 | D-(-)-Quinic acid | C7H12O6 | 191.055 | 0.6086 | 79.4 | neg | 3814068.056 |
| 14 | 9-Oxo-10,12-octadecadienoic acid | C18H30O3 | 295.2262 | 8.362 | 96.4 | pos | 3655684.727 |
| 15 | 5,7,3',4',5'-pentahydroxyflavanone | C15H12O7 | 303.0508 | 4.2025 | 36.3 | neg | 3235131.507 |
| 16 | Antiarol | C9H12O4 | 185.0807 | 3.1739 | 77.3 | pos | 3153149.013 |
| 17 | Nonanedioic acid | C9H16O4 | 187.0967 | 4.7268 | 98.8 | neg | 2606832.103 |
| 18 | Deoxyandrographolide | C20H30O4 | 335.2186 | 11.2549 | 37.5 | pos | 2527576.576 |
| 19 | 5-Hydroxycoumarin | C9H6O3 | 163.0388 | 2.9288 | 93.7 | pos | 2472101.311 |
| 20 | Malic acid | C4H6O5 | 133.013 | 0.6268 | 99.2 | neg | 2411312.49 |
| 21 | Tianshic acid | C18H34O5 | 295.2263 | 10.7455 | 78.9 | pos | 2303631.862 |
| 22 | 2-Pyrrolidinecarboxylic acid | C5H9NO2 | 116.0708 | 0.6144 | 92.9 | pos | 2175747.966 |
| 23 | Isocitrate | C6H8O7 | 191.0189 | 0.6626 | 66.1 | neg | 1987532.031 |
| 24 | 17-Hydroxylinolenic acid | C18H30O3 | 295.2262 | 11.8281 | 95 | pos | 1971086.256 |
| 25 | D-(+)-Lactose | C12H22O11 | 341.1085 | 0.6086 | 91.9 | neg | 1942988.698 |
| 26 | 4-p-Coumaroylquinic acid | C16H18O8 | 337.0928 | 3.3701 | 92.4 | neg | 1819508.564 |
| 27 | 3-(4-Hydroxy-3-methoxyphenyl)propanal | C10H12O3 | 181.0858 | 3.5138 | 83.7 | pos | 1639224.428 |
| 28 | Scopolin | C16H18O9 | 355.1017 | 2.9288 | 64.3 | pos | 1616999.974 |
| 29 | 4-Hydroxyisoleucine | C6H13NO3 | 148.0966 | 0.5607 | 51.2 | pos | 1557020.309 |
| 30 | Adenosine | C10H13N5O4 | 268.1036 | 0.9699 | 89.1 | pos | 1524588.396 |
| 31 | 1-O-Caffeoylquinic acid | C16H18O9 | 707.182 | 2.8973 | 94.7 | neg | 1394882.22 |
| 32 | 2-Isopropylmalic acid | C7H12O5 | 175.0602 | 2.9602 | 98 | neg | 1371640.586 |
| 33 | Stearidonic acid | C18H28O2 | 277.2157 | 8.362 | 85.6 | pos | 1370483.434 |
| 34 | Nandrolone | C18H26O2 | 275.2002 | 11.0771 | 73.7 | pos | 1223918.034 |
| 35 | Rosiridoside B | C21H36O11 | 463.2184 | 3.7239 | 80.2 | neg | 1174311.947 |
| 36 | Sebacic acid | C10H18O4 | 201.1125 | 5.6924 | 97.6 | neg | 1083283.522 |
| 37 | Sarracenin | C11H14O5 | 227.091 | 3.1739 | 50.7 | pos | 963986.9057 |
| 38 | Gluconic acid | C6H12O7 | 195.0501 | 0.5729 | 93 | neg | 927964.4803 |
| 39 | 1,2,3-Trihydroxybenzene | C6H6O3 | 127.0391 | 0.6144 | 76.3 | pos | 905046.4246 |
| 40 | Orcinol glucoside | C13H18O7 | 287.112 | 2.8742 | 57.6 | pos | 869289.4974 |
| 41 | Coronaric acid | C18H32O3 | 297.2419 | 12.289 | 69.7 | pos | 865877.7729 |
| 42 | 5'-Prenyllicodione | C20H20O5 | 341.1377 | 11.8712 | 97.1 | pos | 806684.8075 |
| 43 | Plantagoside | C21H22O12 | 465.1033 | 2.8367 | 80.2 | neg | 806505.548 |
| 44 | 4-(beta-D-glucosyloxy)-3-hydroxy-benzoic acid | C13H16O9 | 315.0721 | 2.0501 | 75.2 | neg | 790307.8619 |
| 45 | Viscidulin I | C15H10O7 | 301.0352 | 5.0401 | 76.1 | neg | 771618.0587 |
| 46 | Neocnidilide | C12H18O2 | 195.1378 | 12.2675 | 77.8 | pos | 715336.5833 |
| 47 | Moracin P | C19H18O5 | 327.1221 | 7.2198 | 79 | pos | 702709.8918 |
| 48 | Aromadendrin | C15H12O6 | 287.056 | 5.5664 | 55.1 | neg | 690767.9262 |
| 49 | Scopoletin | C10H8O4 | 193.0494 | 3.0792 | 97.9 | pos | 686621.5783 |
| 50 | Coumarin | C9H6O2 | 147.044 | 3.3586 | 96 | pos | 643616.4918 |
| 51 | 4-n-Hexylphenol | C12H18O | 179.1428 | 13.1696 | 87.5 | pos | 603025.9389 |
| 52 | L-nmma | C7H16N4O2 | 189.1343 | 0.5788 | 76.7 | pos | 601228.9462 |
| 53 | (+)-Muscarine | C9H19NO2 | 174.1488 | 3.0982 | 47.6 | pos | 575631.8659 |
| 54 | 4-Caffeoylquinic acid | C16H18O9 | 353.0877 | 2.4524 | 91.6 | neg | 559124.7914 |
| 55 | Cis-2,8-Menthadien-1-ol | C10H16O | 153.1273 | 3.7338 | 56.5 | pos | 558470.2506 |
| 56 | Tuberonic acid | C12H18O4 | 225.1127 | 6.1583 | 94 | neg | 554680.0544 |
| 57 | 4-Hydroxy-2-methylacetophenone | C9H10O2 | 151.0753 | 3.2839 | 88.7 | pos | 544528.9658 |
| 58 | Purpurin | C14H8O5 | 255.0296 | 4.3061 | 87.9 | neg | 534190.0234 |
| 59 | Citrinin | C13H14O5 | 251.091 | 2.8742 | 70.7 | pos | 485815.6311 |
| 60 | Isobavachin | C20H20O4 | 323.1285 | 12.6481 | 95 | neg | 477321.7947 |
| 61 | Isoscopoletin | C10H8O4 | 193.0494 | 4.056 | 96.4 | pos | 466878.3637 |
| 62 | Carnosic acid | C20H28O4 | 333.203 | 12.7934 | 35.2 | pos | 454248.088 |
| 63 | 2,6-Dimethoxyphenol | C8H10O3 | 155.0701 | 2.8742 | 73.8 | pos | 445179.5782 |
| 64 | Phaseoloidin | C14H18O9 | 329.0876 | 2.1337 | 63.7 | neg | 444984.4051 |
| 65 | Guanine | C5H5N5O | 152.0566 | 0.9699 | 87.4 | pos | 429522.162 |
| 66 | Traumatic Acid | C12H20O4 | 227.1283 | 7.7413 | 98.1 | neg | 408933.826 |
| 67 | 4-aminobutanoic acid | C4H9NO2 | 104.0711 | 0.5788 | 79.4 | pos | 401268.6175 |
| 68 | Betaine | C5H11NO2 | 118.0864 | 0.5966 | 82.1 | pos | 398688.6254 |
| 69 | Alpha-Sinensal | C15H22O | 219.1741 | 12.8602 | 66.2 | pos | 394731.3443 |
| 70 | Umbelliferone | C9H6O3 | 161.0234 | 3.9934 | 93.9 | neg | 385076.6012 |
| 71 | Succinic acid | C4H6O4 | 117.0181 | 0.9712 | 64.7 | neg | 383069.4005 |
| 72 | Aromatic aldehyde | C7H6O | 107.0496 | 1.9558 | 94.7 | pos | 373212.6237 |
| 73 | Gibberellin A53 | C20H28O5 | 349.1979 | 10.6145 | 54.1 | pos | 364244.4355 |
| 74 | 6-Methyl-7-(3-oxobutyl)-bicyclo[4.1.0]heptan-3-one | C12H18O2 | 195.1378 | 7.42 | 79.7 | pos | 361564.9414 |
| 75 | 2-Phenylethyl formate | C9H10O2 | 151.0753 | 3.5536 | 74.8 | pos | 342165.562 |
| 76 | (-)-gamma-Cuparenol | C15H22O | 273.1844 | 9.7954 | 54.2 | pos | 339773.3713 |
| 77 | Methyl veratrate | C10H12O4 | 197.0807 | 3.1918 | 49 | pos | 330740.3811 |
| 78 | Gentisic acid | C7H6O4 | 153.0182 | 3.1238 | 87.8 | neg | 324928.9015 |
| 79 | Sanggenon H | C20H18O6 | 353.1027 | 10.944 | 45.7 | neg | 319886.4475 |
| 80 | Alpha-D-Glucose | C6H12O6 | 179.0551 | 0.5907 | 70.1 | neg | 319869.2315 |
| 81 | Cinnamaldehyde | C9H8O | 133.0648 | 2.6829 | 71.3 | pos | 318488.269 |
| 82 | 3,4'-Dihydroxypropiophenone | C9H10O3 | 167.0702 | 3.1918 | 72.3 | pos | 307359.9057 |
| 83 | Estriol | C18H24O3 | 289.1794 | 10.3713 | 75.4 | pos | 302043.8049 |
| 84 | Quercetin 3-galactoside | C21H20O12 | 463.088 | 3.6189 | 48 | neg | 301215.3474 |
| 85 | Methylgingerol | C18H28O4 | 309.2054 | 9.7954 | 61.2 | pos | 297528.7835 |
| 86 | Beta-D-Glucosamine | C6H13NO5 | 180.0864 | 0.5966 | 60.1 | pos | 295873.874 |
| 87 | 4-Guanidino-1-butanol | C5H13N3O | 132.1131 | 0.7743 | 62.1 | pos | 295431.3708 |
| 88 | Cnidilide | C12H18O2 | 195.1378 | 9.3585 | 96.4 | pos | 292971.4875 |
| 89 | P-Coumaroyl quinic acid | C16H18O8 | 339.107 | 3.3586 | 91.4 | pos | 292736.268 |
| 90 | Mesaconic acid | C5H6O4 | 129.0181 | 0.8745 | 70.5 | neg | 289378.8421 |
| 91 | Gajutsulactone A | C15H22O2 | 235.1689 | 9.468 | 62.8 | pos | 288787.6586 |
| 92 | (R)-(+)-2-Pyrrolidone-5-carboxylic acid | C5H7NO3 | 130.05 | 0.8632 | 61 | pos | 288366.2901 |
| 93 | 1,1-Dimethylpyrrolidinium-2-carboxylate | C7H13NO2 | 144.1018 | 0.6321 | 36.8 | pos | 285879.2424 |
| 94 | O-Cymol | C10H14 | 135.1168 | 3.7338 | 87.3 | pos | 273743.1846 |
| 95 | Mulberroside A | C26H32O14 | 567.1713 | 2.7513 | 99.6 | neg | 262969.5003 |
| 96 | 5-O-Caffeoylshikimic acid | C16H16O8 | 335.0771 | 3.4545 | 83.4 | neg | 261100.3348 |
| 97 | Isobavachalcone | C20H20O4 | 325.1428 | 12.6616 | 99.1 | pos | 256552.8405 |
| 98 | Lactobionic acid | C12H22O12 | 357.1035 | 0.5729 | 58.4 | neg | 252558.8332 |
| 99 | Apocynin | C9H10O3 | 167.0702 | 2.3418 | 71.2 | pos | 241469.2043 |
| 100 | Benzoic acid | C7H6O2 | 123.0443 | 1.994 | 79.1 | pos | 238710.3139 |
| 101 | Curcumenolactone C | C15H20O4 | 265.1431 | 2.8559 | 66.6 | pos | 238685.1717 |
| 102 | D-Pantothenic acid | C9H17NO5 | 220.1178 | 2.3418 | 92.4 | pos | 237461.4662 |
| 103 | (R)-(+)-Pulegone | C10H16O | 153.1273 | 4.5794 | 83 | pos | 233024.6564 |
| 104 | Isotachioside | C13H18O8 | 301.0926 | 1.4578 | 39.1 | neg | 231505.2761 |
| 105 | Effusanin A | C20H28O5 | 349.198 | 7.4837 | 64.5 | pos | 230854.2214 |
| 106 | Quercetin-7-O-beta-D-glucopyranoside | C21H20O12 | 463.0879 | 4.0148 | 88.8 | neg | 226575.534 |
| 107 | Trans-Aconitate | C6H6O6 | 173.0082 | 0.8745 | 77.7 | neg | 225731.5552 |
| 108 | Ligustilide | C12H14O2 | 191.1065 | 5.2332 | 75 | pos | 225142.8552 |
| 109 | Protocatechuic acid | C7H6O4 | 153.0183 | 2.1742 | 96.3 | neg | 224916.5701 |
| 110 | Dihydromyricetin | C15H12O8 | 303.0495 | 5.0809 | 62.3 | pos | 224522.1049 |
| 111 | Indoline | C8H9N | 120.081 | 1.8598 | 62.4 | pos | 223392.966 |
| 112 | Isoguanosine | C10H13N5O5 | 284.0985 | 0.9699 | 83.4 | pos | 222525.5411 |
| 113 | Jasmonic acid | C12H18O3 | 211.1326 | 12.617 | 98.2 | pos | 221503.5855 |
| 114 | Undecanedioic acid | C11H20O4 | 215.1282 | 7.0387 | 89.6 | neg | 218062.952 |
| 115 | Mulberrin | C25H26O6 | 423.1793 | 12.3109 | 95.6 | pos | 215060.5467 |
| 116 | Ectocarpene | C11H16 | 149.1324 | 8.3216 | 90.3 | pos | 213136.0754 |
| 117 | 7,8-Dihydroxyflavone | C15H10O4 | 287.091 | 8.282 | 37.1 | pos | 208785.4016 |
| 118 | Quercetagetin | C15H10O8 | 317.0301 | 5.082 | 51.5 | neg | 205851.1719 |
| 119 | Ingol | C20H30O6 | 367.2083 | 7.2424 | 50.4 | pos | 203176.7736 |
| 120 | Benzyl acuminose | C18H26O10 | 401.145 | 3.3502 | 82.4 | neg | 194900.0888 |
| 121 | Alternariol | C14H10O5 | 257.0453 | 5.315 | 54.5 | neg | 191486.5082 |
| 122 | 3-O-Feruloylquinic acid | C17H20O9 | 367.1031 | 3.6189 | 97.4 | neg | 187857.2768 |
| 123 | Isodemethylwedelolactone | C15H8O7 | 299.0196 | 5.5874 | 78.2 | neg | 187122.3805 |
| 124 | Hyperoside | C21H20O12 | 465.1023 | 3.6337 | 97.8 | pos | 186897.6806 |
| 125 | Caffeic acid | C9H8O4 | 179.0341 | 3.1446 | 98.4 | neg | 178955.9558 |
| 126 | 5-O-Methylembelin | C18H28O4 | 307.1913 | 12.2818 | 66 | neg | 177100.3345 |
| 127 | Dihydroactinidiolide | C11H16O2 | 181.1221 | 6.1897 | 82.7 | pos | 176479.6877 |
| 128 | Vitedoin A | C20H20O6 | 357.1326 | 6.2119 | 87.3 | pos | 176266.9444 |
| 129 | 2,4,5,6-Tetrahydroxyphenanthrene | C14H10O4 | 243.065 | 4.2172 | 95 | pos | 174686.9261 |
| 130 | 3,4-Dihydroxybenzaldehyde | C7H6O3 | 137.0232 | 2.7513 | 68.3 | neg | 174115.1127 |
| 131 | Skimmin | C15H16O8 | 325.0913 | 2.9475 | 97.9 | pos | 174082.5669 |
| 132 | Isonicotinic acid | C6H5NO2 | 124.0394 | 0.7921 | 55.8 | pos | 169479.7246 |
| 133 | Eriodictyol chalcone | C15H12O6 | 287.0559 | 4.6856 | 87.1 | neg | 169224.7004 |
| 134 | 2-Hydroxy-4-methoxybenzaldehyde | C8H8O3 | 153.0545 | 3.1739 | 77.2 | pos | 166622.3052 |
| 135 | Jioglutin E | C11H20O5 | 231.1233 | 4.5577 | 96.1 | neg | 164216.8299 |
| 136 | DL-2-Aminoadipic acid | C6H11NO4 | 162.0759 | 0.543 | 53.3 | pos | 163480.3438 |
| 137 | 2-Pyrrolidinone | C4H7NO | 86.0607 | 0.5788 | 75 | pos | 162060.357 |
| 138 | Piceatannol | C14H12O4 | 245.0806 | 3.5324 | 85.7 | pos | 161023.1847 |
| 139 | Ibuprofen | C13H18O2 | 207.1378 | 3.456 | 87.2 | pos | 160970.9219 |
| 140 | Momilactone B | C20H26O4 | 331.1874 | 9.2481 | 42.7 | pos | 160593.5452 |
| 141 | 5,7-Dihydroxycoumarin | C9H6O4 | 177.0184 | 3.1238 | 60.6 | neg | 158275.8583 |
| 142 | Methacrylamide | C4H7NO | 86.0607 | 1.0411 | 48.9 | pos | 158045.4434 |
| 143 | Trans-2-Octenal | C8H14O | 125.096 | 4.7268 | 86.8 | neg | 155933.6403 |
| 144 | Xanthoxylin | C10H12O4 | 197.0807 | 2.8742 | 77.2 | pos | 149455.4398 |
| 145 | Dehydroandrographolide | C20H28O4 | 333.203 | 9.5997 | 64.4 | pos | 147369.3441 |
| 146 | Morin | C15H10O7 | 303.0493 | 11.8281 | 86.1 | pos | 146789.0707 |
| 147 | Daphnetin | C9H6O4 | 177.0184 | 4.3669 | 64.3 | neg | 142389.6334 |
| 148 | Liquiritigenin | C15H12O4 | 255.066 | 7.7413 | 73.1 | neg | 137577.3123 |
| 149 | Aurantiamide acetate | C27H28N2O4 | 445.2113 | 11.6943 | 86.6 | pos | 132145.3407 |
| 150 | Demethylwedelolactone | C15H8O7 | 299.0196 | 5.0616 | 88.9 | neg | 131541.7235 |
| 151 | 4-Coumaric acid | C9H8O3 | 163.039 | 3.7653 | 90.8 | neg | 129796.1023 |
| 152 | Trans-Zeatin | C10H13N5O | 220.1178 | 2.1869 | 52.2 | pos | 129538.9398 |
| 153 | Valerophenone | C11H14O | 163.1116 | 5.2332 | 67.8 | pos | 128141.0234 |
| 154 | 5-Hydroxymethyl-2-Furaldehyde | C6H6O3 | 127.0391 | 15.9819 | 91.5 | pos | 127154.3762 |
| 155 | Boschnialactone | C9H14O2 | 155.1065 | 7.4407 | 60.1 | pos | 126540.0611 |
| 156 | 6-Hydroxykaempferol | C15H10O7 | 301.0352 | 4.1613 | 55.8 | neg | 125911.5536 |
| 157 | Dihydrocarveyl acetate | C12H20O2 | 197.1534 | 4.8211 | 75.1 | pos | 122474.4834 |
| 158 | 2-hydroxyhexadecanoic acid | C16H32O3 | 271.2276 | 13.9441 | 98.3 | neg | 122303.3799 |
| 159 | Isovanillic acid | C8H8O4 | 169.0494 | 2.1289 | 88.9 | pos | 120600.6125 |
| 160 | Suberic acid | C8H14O4 | 173.081 | 3.9729 | 87.3 | neg | 120379.7308 |
| 161 | Confertifoline | C15H22O2 | 235.1689 | 7.7241 | 84.5 | pos | 118079.0567 |
| 162 | Apiopaeonoside | C20H28O12 | 459.1503 | 2.859 | 45.8 | neg | 117609.5996 |
| 163 | Kuwanon G | C40H36O11 | 691.218 | 11.7841 | 94.4 | neg | 114908.8863 |
| 164 | 1-Kestose | C18H32O16 | 503.1614 | 0.6268 | 96.7 | neg | 113440.1731 |
| 165 | Syringic acid | C9H10O5 | 199.06 | 2.4 | 94.8 | pos | 112452.7615 |
| 166 | Licoflavonol | C20H18O6 | 355.1169 | 10.9443 | 88.5 | pos | 111082.8024 |
| 167 | Sesamin | C20H18O6 | 387.1431 | 6.6796 | 37.2 | pos | 110807.6472 |
| 168 | Isokobusone | C14H22O2 | 223.169 | 9.2036 | 53.5 | pos | 109998.3551 |
| 169 | Alantolactone | C15H20O2 | 233.1533 | 11.0332 | 81.9 | pos | 109932.2326 |
| 170 | Isosinensetin | C20H20O7 | 355.1169 | 11.6943 | 45 | pos | 109300.8874 |
| 171 | Furfural | C5H4O2 | 97.0289 | 0.6144 | 45.1 | pos | 109169.2556 |
| 172 | Dictamnol | C12H18O | 179.1429 | 4.8211 | 78.9 | pos | 108149.0192 |
| 173 | L-Asparagine | C4H8N2O3 | 131.045 | 0.5542 | 85.3 | neg | 107729.1927 |
| 174 | Itaconic acid | C5H6O4 | 129.0181 | 0.6626 | 51 | neg | 106134.6652 |
| 175 | Spiraeoside | C21H20O12 | 465.1023 | 4.0156 | 96.5 | pos | 105917.7704 |
| 176 | 4-Methylumbelliferone | C10H8O3 | 177.0545 | 3.6337 | 94.4 | pos | 105394.7583 |
| 177 | 3,5,7,2',6'-Pentahydroxyflavanone | C15H12O7 | 305.0651 | 2.8364 | 87 | pos | 104413.7143 |
| 178 | Dibutyl phthalate | C16H22O4 | 279.1586 | 13.3703 | 61.9 | pos | 104349.8901 |
| 179 | Anemonin | C10H8O4 | 191.0341 | 4.0563 | 38.7 | neg | 99235.81777 |
| 180 | (S)-5-Hydroxymethyl-2[5H]-furanone | C5H6O3 | 115.0392 | 2.8742 | 38.3 | pos | 98395.99096 |
| 181 | Vanillic acid | C8H8O4 | 167.034 | 2.1337 | 98.3 | neg | 98204.80659 |
| 182 | Moracin N | C19H18O4 | 311.1274 | 10.527 | 85.4 | pos | 96588.82554 |
| 183 | Osmanthuside H | C19H28O11 | 431.1555 | 3.7025 | 71.9 | neg | 94995.21788 |
| 184 | Eucommiol | C9H16O4 | 171.1014 | 12.1148 | 45.9 | pos | 93878.55373 |
| 185 | Isoferulic acid | C10H10O4 | 177.0545 | 4.6667 | 80.5 | pos | 93184.49659 |
| 186 | Ricinoleic acid | C18H34O3 | 297.243 | 13.36 | 53 | neg | 92504.52506 |
| 187 | Paeonolide | C20H28O12 | 459.1505 | 2.4738 | 75.7 | neg | 89677.87592 |
| 188 | Sedanonic acid | C12H18O3 | 211.1326 | 9.2481 | 66.2 | pos | 86855.48192 |
| 189 | 8-Formyl-5-O-methylophiopogonanone B | C20H20O6 | 355.1184 | 9.781 | 42.9 | neg | 85095.90306 |
| 190 | Estrone | C18H22O2 | 271.1689 | 10.6145 | 66.4 | pos | 84419.35018 |
| 191 | Trans-Cinnamate | C9H8O2 | 149.0597 | 3.0606 | 71.1 | pos | 84225.88496 |
| 192 | Oxyphyllenodiol B | C14H22O3 | 237.1491 | 10.1332 | 38.8 | neg | 84023.62752 |
| 193 | Corchorifatty acid F | C18H32O5 | 311.221 | 7.42 | 85.2 | pos | 83705.74853 |
| 194 | Guanosine | C10H13N5O5 | 282.0841 | 0.9909 | 92.9 | neg | 83699.6135 |
| 195 | Uridine | C9H12N2O6 | 243.0618 | 0.8745 | 96.6 | neg | 82599.75389 |
| 196 | Tuberonic acid glucoside | C18H28O9 | 387.166 | 3.3912 | 52.4 | neg | 82555.30463 |
| 197 | 1-O-Galloyl-beta-D-glucose | C13H16O10 | 331.0669 | 1.0688 | 56.3 | neg | 82507.96433 |
| 198 | Isoliquiritigenin | C15H12O4 | 257.0805 | 3.6137 | 68.9 | pos | 82345.15781 |
| 199 | 13-Apo-beta-carotenone | C18H26O | 259.2053 | 12.7716 | 93.5 | pos | 81827.13282 |
| 200 | Luteolin | C15H10O6 | 285.0402 | 4.1613 | 83.5 | neg | 80981.92307 |
| 201 | 5,7-Dihydroxychromone | C9H6O4 | 177.0184 | 3.5987 | 77.7 | neg | 80397.87445 |
| 202 | 5-Hydroxymaltol | C6H6O4 | 143.0339 | 1.6035 | 38.9 | pos | 80040.53584 |
| 203 | Kobusone | C14H22O2 | 223.169 | 10.3928 | 68.8 | pos | 79811.56258 |
| 204 | 2-Methylbenzoic acid | C8H8O2 | 135.044 | 3.1446 | 48.9 | neg | 79174.78591 |
| 205 | Cytosine | C4H5N3O | 112.0509 | 0.6144 | 37.9 | pos | 79160.41815 |
| 206 | Emodin anthrone | C15H12O4 | 255.066 | 8.8848 | 82.9 | neg | 76862.82201 |
| 207 | Benzene-1,2,4-triol | C6H6O3 | 144.0655 | 1.3327 | 49.8 | pos | 76322.11392 |
| 208 | Nicotinamide | C6H6N2O | 123.0554 | 0.7921 | 70.9 | pos | 76110.45296 |
| 209 | Arctigenin | C21H24O6 | 373.1639 | 7.5923 | 60.2 | pos | 75965.44558 |
| 210 | Ajugol | C15H24O9 | 313.1276 | 3.5536 | 51.4 | pos | 74339.11215 |
| 211 | Ethylbenzene | C8H10 | 107.0859 | 3.7338 | 89.5 | pos | 72425.43854 |
| 212 | P-hydroxylphenylpropanol | C9H10O3 | 147.044 | 4.3258 | 40 | neg | 71202.17587 |
| 213 | 2,6-Dimethoxy-benzoic acid | C9H10O4 | 183.0651 | 2.457 | 56.5 | pos | 70580.50511 |
| 214 | Catalpol | C15H22O10 | 343.1034 | 2.5169 | 40.1 | neg | 68212.34185 |
| 215 | Piceatannol 3'-O-glucoside | C20H22O9 | 407.133 | 2.7792 | 92.1 | pos | 67228.227 |
| 216 | Icosatrienoic acid | C20H34O2 | 324.2891 | 13.6282 | 63.2 | pos | 67199.46932 |
| 217 | Rosavin | C20H28O10 | 427.1604 | 4.3061 | 89.6 | neg | 66784.17044 |
| 218 | Qing Hau Sau | C15H22O5 | 283.153 | 5.5654 | 94.2 | pos | 65671.78569 |
| 219 | Caffeic Acid Methyl Ester | C10H10O4 | 195.0651 | 2.5896 | 69.2 | pos | 65548.66108 |
| 220 | 4-n-Pentylphenol | C11H16O | 165.1273 | 6.6574 | 91 | pos | 65080.08698 |
| 221 | Astringin | C20H22O9 | 405.119 | 3.5365 | 94.7 | neg | 64669.52294 |
| 222 | Pseudolaroside A | C13H16O8 | 299.077 | 1.6039 | 85.2 | neg | 64550.06913 |
| 223 | (2R)-2-(3,4-Dihydroxyphenyl)-3,5,7-trihydroxy-2,3-dihydrochromen-4-one | C15H12O7 | 303.0508 | 2.8367 | 98.3 | neg | 64523.63431 |
| 224 | L-Phenylalanine | C9H11NO2 | 166.0862 | 1.8407 | 98.6 | pos | 64044.43251 |
| 225 | Achillin | C15H18O3 | 247.1326 | 5.6322 | 44.9 | pos | 63952.98707 |
| 226 | 3,8-dihydroxy-urolithin | C13H8O4 | 273.0403 | 3.745 | 44.9 | neg | 63787.11739 |
| 227 | Aesculetin | C9H6O4 | 177.0183 | 2.6673 | 92.1 | neg | 62966.39325 |
| 228 | Sedanonic acid lactone | C12H16O2 | 193.1221 | 12.2675 | 79.6 | pos | 61197.39764 |
| 229 | Roughanic acid | C16H26O2 | 251.2002 | 12.2675 | 83.1 | pos | 61141.15595 |
| 230 | P-Cymene | C10H14 | 135.1167 | 12.7716 | 58.4 | pos | 60522.33192 |
| 231 | Methyl 4-hydroxybenzoate | C8H8O3 | 153.0546 | 3.8128 | 58.7 | pos | 60340.81539 |
| 232 | 4-hydroxy-benzoate | C7H6O3 | 137.0233 | 4.8735 | 92 | neg | 59832.99729 |
| 233 | 2-Hydroxycinnamaldehyde | C9H8O2 | 149.0597 | 1.2036 | 56.7 | pos | 59775.30784 |
| 234 | Farnesyl acetate | C17H28O2 | 265.2158 | 11.5828 | 78.9 | pos | 59725.0307 |
| 235 | Oxyresveratrol | C14H12O4 | 245.0806 | 2.7792 | 97.3 | pos | 59555.32995 |
| 236 | Magnolone | C21H22O7 | 387.1431 | 7.0846 | 86.9 | pos | 59227.12357 |
| 237 | Grasshopper ketone | C13H20O3 | 225.1483 | 4.0156 | 76.4 | pos | 58976.96676 |
| 238 | Gamma-Methoxyisoeugenol | C11H14O3 | 195.1015 | 3.3032 | 84.3 | pos | 58618.7361 |
| 239 | D-Mannoheptulose | C7H14O7 | 209.0659 | 0.6086 | 82.1 | neg | 58607.29654 |
| 240 | 3-Hydroxymorindone | C15H10O6 | 285.0403 | 5.6087 | 55.1 | neg | 58307.7382 |
| 241 | Emodin | C15H10O5 | 271.0596 | 8.282 | 44.4 | pos | 58263.86326 |
| 242 | Androsin | C15H20O8 | 327.1084 | 2.5379 | 80.9 | neg | 58036.91806 |
| 243 | Inosine | C10H12N4O5 | 267.0723 | 1.0115 | 64.3 | neg | 57950.54025 |
| 244 | Rishitin | C14H22O2 | 223.1689 | 13.0367 | 89.9 | pos | 57868.2232 |
| 245 | Caffeic acid 3-glucoside | C15H18O9 | 341.0877 | 2.4524 | 57.8 | neg | 57521.71474 |
| 246 | Camphor | C10H16O | 153.1272 | 0.3866 | 87.3 | pos | 57483.6528 |
| 247 | 9-Oxononanoic acid | C9H16O3 | 171.1017 | 6.0948 | 90.9 | neg | 56922.17153 |
| 248 | 2-keto-D-Gluconic acid | C6H10O7 | 193.0344 | 0.6086 | 57.1 | neg | 56419.56174 |
| 249 | Polydatin | C20H22O8 | 391.138 | 2.8921 | 88.2 | pos | 56025.41014 |
| 250 | 2'-O-Methyladenosine | C11H15N5O4 | 282.1193 | 1.5252 | 70.9 | pos | 54695.22151 |
| 251 | Rosarin | C20H28O10 | 427.1605 | 4.664 | 82.2 | neg | 54118.49743 |
| 252 | Bergaptol I | C20H26O11 | 443.1589 | 2.0715 | 48.9 | pos | 53808.97339 |
| 253 | 4-Hydroxycinnamyl aldehyde | C9H8O2 | 149.0597 | 1.8598 | 83.5 | pos | 53752.68623 |
| 254 | (+/-)-Tryptophan | C11H12N2O2 | 205.097 | 2.5709 | 99.2 | pos | 53624.13937 |
| 255 | Kushenol F | C25H28O6 | 407.1845 | 13.1696 | 51 | pos | 53171.43124 |
| 256 | Dihydroartemisinic acid | C15H24O2 | 237.1846 | 11.2549 | 47 | pos | 52534.57374 |
| 257 | Ilicic acid | C15H24O3 | 235.169 | 10.3488 | 59.3 | pos | 52140.17159 |
| 258 | Senkyunolide H | C12H16O4 | 225.1118 | 7.3094 | 75.3 | pos | 52023.68323 |
| 259 | Linderane | C15H16O4 | 261.1118 | 5.8553 | 57.2 | pos | 50627.98722 |
| 260 | Calystegine B1 | C7H13NO4 | 158.0811 | 2.1682 | 43.1 | pos | 50490.78535 |
| 261 | Estradiol | C18H24O2 | 273.1845 | 10.6145 | 50.4 | pos | 50152.52193 |
| 262 | Zingerone | C11H14O3 | 195.1015 | 3.5936 | 79.3 | pos | 49331.6995 |
| 263 | Beta-Mangostin | C25H28O6 | 425.1956 | 12.617 | 56.8 | pos | 49243.78133 |
| 264 | P-Mentha-1,3,8-triene | C10H14 | 135.1168 | 6.1897 | 72.4 | pos | 49072.65207 |
| 265 | Crocusatin B | C10H16O3 | 183.1018 | 5.6924 | 35.2 | neg | 48362.35925 |
| 266 | Paeonoside | C15H20O8 | 327.1084 | 2.7725 | 93 | neg | 48289.84456 |
| 267 | Atractyloside A | C21H36O10 | 447.2234 | 5.987 | 93.6 | neg | 48132.31853 |
| 268 | Hydroquinone | C6H6O2 | 109.0282 | 2.1742 | 80.1 | neg | 47963.97904 |
| 269 | Fa(7:0) | C7H14O2 | 189.1124 | 4.664 | 37.3 | neg | 47900.20469 |
| 270 | 5-Methoxysalicylic acid | C8H8O4 | 167.034 | 3.2055 | 97.6 | neg | 47770.19395 |
| 271 | N,N'-Dicyclohexylurea | C13H24N2O | 225.1958 | 9.2922 | 51.3 | pos | 47253.87451 |
| 272 | Butin | C15H12O5 | 271.061 | 4.7064 | 57.1 | neg | 45426.09471 |
| 273 | Sinapyl alcohol | C11H14O4 | 211.0963 | 1.994 | 92.2 | pos | 44389.78872 |
| 274 | Perillene | C10H14O | 151.1116 | 12.2675 | 88.2 | pos | 44259.85915 |
| 275 | 4,2',4',alpha-Tetrahydroxydihydrochalcone | C15H14O5 | 257.0804 | 7.8137 | 40.5 | pos | 43806.31002 |
| 276 | Cytidine | C9H13N3O5 | 244.0925 | 0.6144 | 73 | pos | 42724.6005 |
| 277 | Nepodin | C13H12O3 | 215.0707 | 9.9572 | 46.3 | neg | 42704.95394 |
| 278 | Isorhamnetin | C16H12O7 | 315.051 | 5.8589 | 90.6 | neg | 42563.17482 |
| 279 | Vanillin | C8H8O3 | 151.039 | 3.8263 | 97.7 | neg | 42469.3852 |
| 280 | 4-Isopropylbenzaldehyde | C10H12O | 149.0961 | 3.456 | 81 | pos | 42160.87778 |
| 281 | Anethole | C10H12O | 149.0961 | 3.7736 | 60.8 | pos | 41399.41395 |
| 282 | 4-(beta-D-Glucopyranosyloxy)-3-methoxybenzoic acid | C14H18O9 | 348.1284 | 2.1289 | 63.9 | pos | 40742.67061 |
| 283 | Panaxjapyne C | C17H26O3 | 279.1951 | 10.8116 | 60.6 | pos | 40105.16803 |
| 284 | Adenine | C5H5N5 | 134.046 | 1.2298 | 68.6 | neg | 40082.35108 |
| 285 | (+)-Nootkatone | C15H22O | 219.1741 | 3.9541 | 75.4 | pos | 40076.8514 |
| 286 | Parishin E | C19H24O13 | 459.114 | 2.7302 | 69.2 | neg | 39727.03387 |
| 287 | Chavicol | C9H10O | 135.0804 | 3.0606 | 82.7 | pos | 39472.55732 |
| 288 | Scoparone | C11H10O4 | 207.0651 | 4.9495 | 97.5 | pos | 39397.91822 |
| 289 | 2-Hydroxycinnamic acid | C9H8O3 | 165.0545 | 0.8988 | 77.9 | pos | 39298.28109 |
| 290 | Fabiatrin | C21H26O13 | 487.1439 | 3.0792 | 86.5 | pos | 39147.21665 |
| 291 | Licoagroside D | C22H24O10 | 493.135 | 5.4177 | 76.4 | neg | 39141.83983 |
| 292 | Kuwanon A | C25H24O6 | 421.1637 | 12.8602 | 94.4 | pos | 39124.4674 |
| 293 | Damnacanthol | C16H12O5 | 285.0753 | 11.8712 | 72.5 | pos | 39109.22502 |
| 294 | Chrysophanol | C15H10O4 | 287.091 | 5.4096 | 39.6 | pos | 39096.7672 |
| 295 | Guaiacol | C7H8O2 | 125.0598 | 3.1918 | 55.8 | pos | 38759.05231 |
| 296 | 7-Methylcoumarin | C10H8O2 | 161.0596 | 1.8598 | 44.5 | pos | 38195.10958 |
| 297 | Amurensin | C26H30O12 | 533.166 | 4.1825 | 42 | neg | 37437.57544 |
| 298 | Epomediol | C10H18O3 | 231.1233 | 4.9772 | 60.4 | neg | 37181.57161 |
| 299 | Geranic acid | C10H16O2 | 169.1222 | 3.713 | 55 | pos | 37010.60444 |
| 300 | Daidzein | C15H10O4 | 255.0647 | 11.8281 | 82.2 | pos | 36347.14295 |
| 301 | 17-Hydroxyisolathyrol | C20H30O5 | 395.2048 | 9.2154 | 44.3 | neg | 36053.46255 |
| 302 | Kaempferol | C15H10O6 | 285.0403 | 6.7524 | 76.4 | neg | 35801.01208 |
| 303 | Quercetin | C15H10O7 | 303.0493 | 12.7273 | 84.1 | pos | 34847.78786 |
| 304 | Leonuriside A | C14H20O9 | 333.1175 | 2.5709 | 63 | pos | 34749.16595 |
| 305 | Alpha-Cyclogeraniol acetate | C13H22O2 | 211.169 | 3.8723 | 82.3 | pos | 34558.46981 |
| 306 | Senkyunolide | C12H16O2 | 193.1222 | 12.8818 | 52.5 | pos | 34281.59831 |
| 307 | Beta-Hydroxypropiovanillone | C10H12O4 | 197.0808 | 2.5526 | 82.2 | pos | 34264.20515 |
| 308 | 4-Hydroxybenzaldehyde | C7H6O2 | 123.0443 | 2.5335 | 83 | pos | 33623.41876 |
| 309 | Gomisin R | C22H24O7 | 401.1588 | 5.9002 | 89 | pos | 33404.69245 |
| 310 | Cuminyl acetate | C12H16O2 | 193.1222 | 6.6574 | 68.7 | pos | 33188.68491 |
| 311 | Adipic acid | C6H10O4 | 145.0495 | 1.2711 | 35.9 | neg | 33137.61098 |
| 312 | P-hydroxylbenzyl alcohol | C7H8O2 | 107.0495 | 2.9665 | 40.2 | pos | 33025.46241 |
| 313 | (+)-Nortrachelogenin | C20H22O7 | 433.15 | 4.9362 | 57 | neg | 32470.45802 |
| 314 | 3,5-Dimethoxy-4-hydroxybenzaldehyde | C9H10O4 | 181.0497 | 3.9934 | 90.8 | neg | 32356.20062 |
| 315 | 1-O-Feruloyl-beta-D-glucose | C16H20O9 | 355.1032 | 2.7513 | 69.1 | neg | 32168.28891 |
| 316 | 2-Ethyltoluene | C9H12 | 121.1014 | 4.2172 | 88.5 | pos | 31933.97315 |
| 317 | Hypoxanthine | C5H4N4O | 137.0457 | 0.7921 | 49.5 | pos | 31877.12142 |
| 318 | Acetyleugenol | C12H14O3 | 207.1014 | 7.3316 | 55.4 | pos | 31307.70425 |
| 319 | Tetralin | C10H12 | 133.1011 | 8.3216 | 94.3 | pos | 31160.70425 |
| 320 | 2,6,6-Trimethyl-2-cyclohexene-1,4-dione | C9H12O2 | 153.0909 | 3.136 | 66 | pos | 31011.27375 |
| 321 | 2-Octenoic acid | C8H14O2 | 143.1066 | 2.6456 | 41 | pos | 30724.30377 |
| 322 | Brosimacutin D | C20H20O5 | 341.1378 | 10.0138 | 77.4 | pos | 30614.54467 |
| 323 | Alpha-Methylstyrene | C9H10 | 119.0856 | 12.093 | 88.8 | pos | 30253.27555 |
| 324 | D-Sorbitol | C6H14O6 | 181.0708 | 0.5729 | 91.9 | neg | 30239.5501 |
| 325 | Shikimic acid | C7H10O5 | 173.0446 | 3.0219 | 66.4 | neg | 30105.28573 |
| 326 | Longicamphenylone | C14H22O | 207.1741 | 11.2549 | 82.5 | pos | 29942.31961 |
| 327 | Sesamolinol | C20H20O7 | 373.1275 | 4.1758 | 72.9 | pos | 29603.43973 |
| 328 | Jasmone | C11H16O | 165.1272 | 11.8933 | 73.4 | pos | 29448.11253 |
| 329 | Moracin D | C19H16O4 | 309.1118 | 11.2549 | 73.8 | pos | 29271.50733 |
| 330 | Eseramine | C16H22N4O3 | 317.1604 | 4.5356 | 90.7 | neg | 28889.71137 |
| 331 | Xanthatin | C15H18O3 | 247.1326 | 2.8559 | 69 | pos | 28459.21855 |
| 332 | Ferulic acid | C10H10O4 | 193.0498 | 3.4955 | 99.1 | neg | 28248.83223 |
| 333 | Abietic acid | C20H30O2 | 320.258 | 11.0771 | 35.2 | pos | 28065.78095 |
| 334 | Pseudoionone | C13H20O | 193.1586 | 4.5575 | 91.7 | pos | 27937.00093 |
| 335 | 2,3-Dihydroxy-1-(4-hydroxy-3,5-dimethoxyphenyl)-1-propanone | C11H14O6 | 243.0861 | 1.7626 | 37.5 | pos | 27844.02993 |
| 336 | Oxyhydrastinine | C11H11NO3 | 206.0811 | 3.913 | 36.7 | pos | 27833.48648 |
| 337 | Ferulaldehyde | C10H10O3 | 179.0702 | 4.6221 | 82 | pos | 27781.51618 |
| 338 | Annuionone E | C13H22O3 | 225.1491 | 5.1025 | 74.3 | neg | 27112.17822 |
| 339 | Isosteviol | C20H30O3 | 363.215 | 13.055 | 43 | neg | 27025.75108 |
| 340 | Xylobiose | C10H18O9 | 281.0874 | 0.5729 | 49.9 | neg | 26958.1804 |
| 341 | Pc(16:0/0:0) | C24H50NO7P | 496.339 | 13.2807 | 97.1 | pos | 26944.13752 |
| 342 | Rengyoside B | C14H24O8 | 319.1395 | 3.3083 | 76.8 | neg | 26797.08859 |
| 343 | Liquiritin | C21H22O9 | 417.119 | 6.2459 | 75.8 | neg | 26730.23539 |
| 344 | Methyl orsellinate | C9H10O4 | 181.0498 | 2.0915 | 93.6 | neg | 26441.70768 |
| 345 | 3-(3-Hydroxyphenyl)propanoic acid | C9H10O3 | 165.0547 | 2.5379 | 78.4 | neg | 26264.03324 |
| 346 | Cleroindicin B | C8H14O3 | 203.0917 | 3.556 | 61 | neg | 26215.52556 |
| 347 | Acetophenone | C8H8O | 121.065 | 3.713 | 55.1 | pos | 26111.62708 |
| 348 | 1-Isopropyl-3-methylbenzene | C10H14 | 135.1167 | 8.3216 | 39.8 | pos | 25892.91779 |
| 349 | Schizonepetoside E | C16H28O8 | 393.1765 | 3.9516 | 49.2 | neg | 25762.17976 |
| 350 | Methyl 4-hydroxycinnamate | C10H10O3 | 177.0548 | 4.6005 | 42.3 | neg | 25506.06608 |
| 351 | 6-Hydroxynicotinic acid | C6H5NO3 | 138.0185 | 0.9337 | 68.5 | neg | 25485.10952 |
| 352 | Capillanol | C12H14O | 175.1118 | 6.6574 | 73.9 | pos | 24948.71706 |
| 353 | Geranylacetone | C13H22O | 195.1742 | 4.2172 | 84.3 | pos | 24937.60196 |
| 354 | L-Hydroxyproline | C5H9NO3 | 114.0552 | 0.6321 | 43 | pos | 24918.0382 |
| 355 | Dihydroartemisinin | C15H24O5 | 283.1549 | 7.4107 | 65.9 | neg | 24557.31352 |
| 356 | Verbenone | C10H14O | 151.1116 | 10.6145 | 43.3 | pos | 24513.25877 |
| 357 | Nortricycloekasantalic acid | C11H16O2 | 181.1221 | 11.5828 | 67.8 | pos | 24497.04074 |
| 358 | Methyl-4-O-methylgallate | C9H10O5 | 197.0448 | 2.4091 | 92.9 | neg | 24380.21734 |
| 359 | 3,4-Dimethylstyrene | C10H12 | 133.1011 | 12.2675 | 66.8 | pos | 24317.60951 |
| 360 | Cis-Resveratrol | C14H12O3 | 229.0857 | 2.8921 | 92 | pos | 24071.50861 |
| 361 | Butein | C15H12O5 | 273.0754 | 4.7317 | 55 | pos | 23950.09647 |
| 362 | Gibberellin A24 | C20H26O5 | 347.1823 | 8.1297 | 49.7 | pos | 23891.01058 |
| 363 | Melazolide A | C11H16O4 | 195.1015 | 5.0378 | 47.1 | pos | 23785.62629 |
| 364 | Lupinalbin A | C15H8O6 | 317.0652 | 5.8777 | 81.9 | pos | 23729.8193 |
| 365 | 5,7,4'-Trihydroxy-8-methylflavanone | C16H14O5 | 269.0805 | 12.6616 | 73.4 | pos | 23285.07066 |
| 366 | Stemanthrene A | C17H18O4 | 287.125 | 5.6322 | 50 | pos | 23247.29728 |
| 367 | Arachidonic Acid (peroxide free) | C20H32O2 | 305.2469 | 13.5681 | 37.9 | pos | 23047.24468 |
| 368 | 5-Acetylsalicylic acid | C9H8O4 | 181.0494 | 2.4 | 43.5 | pos | 22958.20696 |
| 369 | Licoleafol | C20H20O7 | 371.1134 | 5.6496 | 43.7 | neg | 22933.36096 |
| 370 | Sesamol | C7H6O3 | 121.0286 | 2.9288 | 54.8 | pos | 22906.12855 |
| 371 | L-Threonine | C4H9NO3 | 84.045 | 0.8632 | 39.5 | pos | 22848.0332 |
| 372 | C-veratroylglycol | C10H12O5 | 211.0606 | 2.4738 | 88.2 | neg | 22433.87095 |
| 373 | Albuterol | C13H21NO3 | 240.1591 | 5.7448 | 74.9 | pos | 22165.18602 |
| 374 | DL-Glutamate | C5H9NO4 | 146.0447 | 0.5729 | 90.7 | neg | 22094.4325 |
| 375 | L-Arabinose | C5H10O5 | 149.0444 | 0.6086 | 92.5 | neg | 21942.7732 |
| 376 | Purpurogallin | C11H8O5 | 219.0292 | 2.7097 | 67.8 | neg | 21908.72177 |
| 377 | 2-Adamantanone | C10H14O | 151.1116 | 11.8933 | 53.5 | pos | 21877.44359 |
| 378 | 2-Phenylacetamide | C8H9NO | 136.0756 | 0.8988 | 95.9 | pos | 21674.4967 |
| 379 | Curcumol | C15H24O2 | 237.1847 | 3.9541 | 87.3 | pos | 21665.38839 |
| 380 | Licoflavone C | C20H18O5 | 337.1079 | 10.7469 | 65.9 | neg | 21660.28104 |
| 381 | Bicyclo[4.2.0]octa-1,3,5-triene | C8H8 | 105.0703 | 2.3619 | 95 | pos | 21579.93226 |
| 382 | Pheophorbide a | C35H36N4O5 | 593.2749 | 14.1462 | 81.3 | pos | 21556.98105 |
| 383 | 6-Hydroxyrubiadin | C15H10O5 | 269.0452 | 8.2477 | 64.5 | neg | 21429.72192 |
| 384 | 3-hydroxy-3-methyl-Glutaric acid | C6H10O5 | 323.0981 | 0.9712 | 50.2 | neg | 21352.91721 |
| 385 | Aristolone | C15H22O | 201.1636 | 4.6885 | 37.7 | pos | 21072.17896 |
| 386 | 15-Hydroxydehydroabietic acid | C20H28O3 | 361.1993 | 12.755 | 41.5 | neg | 20741.78045 |
| 387 | Pimelic acid | C7H12O4 | 159.0652 | 3.2055 | 88.2 | neg | 20456.13879 |
| 388 | Xylitol | C5H12O5 | 151.06 | 0.5907 | 85.2 | neg | 20409.42034 |
| 389 | Isopeonol | C9H10O3 | 165.0547 | 3.2676 | 62.9 | neg | 20350.37002 |
| 390 | L-Aspartic Acid | C4H7NO4 | 132.029 | 0.5729 | 83.8 | neg | 20241.01035 |
| 391 | Alpha-Linolenic acid | C18H30O2 | 277.2171 | 13.0764 | 67.6 | neg | 19646.8213 |
| 392 | DL-Pipecolinic acid | C6H11NO2 | 147.1127 | 0.5074 | 85.1 | pos | 19627.9972 |
| 393 | Sweroside | C16H22O9 | 357.1189 | 3.0412 | 81.8 | neg | 19473.69681 |
| 394 | Gallic acid | C7H6O5 | 169.0133 | 1.1276 | 95.5 | neg | 19167.26055 |
| 395 | Crocusatin G | C12H20O2 | 195.1382 | 8.8848 | 47.3 | neg | 19072.7738 |
| 396 | Methyl cinnamate | C10H10O2 | 163.0751 | 2.8559 | 65.2 | pos | 18511.42368 |
| 397 | L-Noradrenaline | C8H11NO3 | 170.0811 | 2.2068 | 54.3 | pos | 18461.46925 |
| 398 | 9,10-Dihydroxystearate | C18H36O4 | 315.2538 | 12.7763 | 90.2 | neg | 18241.25355 |
| 399 | Dehydrolindestrenolide | C15H16O2 | 229.1223 | 5.6102 | 70.7 | pos | 18015.18845 |
| 400 | Picein | C14H18O7 | 297.0977 | 1.2089 | 65.4 | neg | 17988.91106 |
| 401 | Licochalcone B | C16H14O5 | 285.0766 | 9.2593 | 72.9 | neg | 17940.69461 |
| 402 | L-Isoleucine | C6H13NO2 | 132.102 | 3.0236 | 75.3 | pos | 17810.79095 |
| 403 | Santene | C9H14 | 123.1169 | 12.4194 | 80.1 | pos | 17653.46923 |
| 404 | (-)-Carvone | C10H14O | 151.1117 | 4.5794 | 83.3 | pos | 17530.99955 |
| 405 | (-)-Glyceollin I | C20H18O5 | 337.108 | 6.1583 | 93.6 | neg | 17492.73191 |
| 406 | 4-Methylcatechol | C7H8O2 | 125.0599 | 2.1289 | 71 | pos | 17400.89986 |
| 407 | Blumenol B | C13H22O3 | 227.1638 | 11.2549 | 67.8 | pos | 17390.39798 |
| 408 | 5,7-Dihydroxyisoflavone | C15H10O4 | 255.0647 | 5.2771 | 65.5 | pos | 17304.44925 |
| 409 | Ethyl 4-methoxycinnamate | C12H14O3 | 207.1014 | 4.3195 | 66.8 | pos | 17177.47114 |
| 410 | 5,7-Dihydroxyflavone | C15H10O4 | 253.0504 | 6.2682 | 70.1 | neg | 17098.1209 |
| 411 | Glutarate | C5H8O4 | 133.0495 | 1.9745 | 47.2 | pos | 17084.15174 |
| 412 | Pentadecanal | C15H30O | 271.2277 | 12.861 | 37.3 | neg | 17031.81271 |
| 413 | Hydroxytyrosol | C8H10O3 | 153.0545 | 2.0501 | 71 | neg | 16954.47867 |
| 414 | Micheliolide | C15H20O3 | 249.1482 | 10.9889 | 35.9 | pos | 16897.89973 |
| 415 | Artemisinic acid | C15H22O2 | 235.169 | 3.8319 | 81.1 | pos | 16748.44774 |
| 416 | Pyrocatechol | C6H6O2 | 109.0282 | 1.375 | 47.4 | neg | 16730.1406 |
| 417 | Isovanillin | C8H8O3 | 151.039 | 2.5169 | 77.3 | neg | 16251.03511 |
| 418 | Shinjulactone C | C20H22O7 | 375.1432 | 4.7097 | 46.7 | pos | 16175.34673 |
| 419 | (S)-(+)-alpha-Phellandrene | C10H16 | 137.1324 | 13.0367 | 78.4 | pos | 16097.75193 |
| 420 | Sorbic acid | C6H8O2 | 113.06 | 2.5896 | 63.6 | pos | 15902.09598 |
| 421 | Melilotoside | C15H18O8 | 325.0928 | 2.5169 | 83.2 | neg | 15708.84898 |
| 422 | 4-Acetyl-1-methylcyclohexene | C9H14O | 139.1117 | 4.9066 | 64.9 | pos | 15672.91358 |
| 423 | (-)-Abscisic acid | C15H20O4 | 263.1287 | 5.5874 | 96.3 | neg | 15597.54505 |
| 424 | Methylnissolin | C17H16O5 | 301.1065 | 9.9919 | 43.4 | pos | 15455.07879 |
| 425 | Nonanoic acid | C9H18O2 | 159.1378 | 5.0157 | 39.9 | pos | 15150.18182 |
| 426 | Phytosphingosine | C18H39NO3 | 318.2997 | 10.5713 | 95.2 | pos | 15128.92328 |
| 427 | Herniarin | C10H8O3 | 177.0545 | 3.0792 | 84.8 | pos | 15028.37279 |
| 428 | Isochlorogenic acid C | C25H24O12 | 515.1191 | 4.5356 | 97.4 | neg | 14803.09707 |
| 429 | Esculin | C15H16O9 | 339.0719 | 2.1543 | 94.9 | neg | 14787.82655 |
| 430 | Ingenol | C20H28O5 | 393.189 | 7.4552 | 41.1 | neg | 14765.34571 |
| 431 | Euparin | C13H12O3 | 215.0707 | 10.3987 | 44.2 | neg | 14741.57549 |
| 432 | 3,5-Dicaffeoylquinic acid | C25H24O12 | 515.119 | 4.4083 | 94.9 | neg | 14723.51981 |
| 433 | L-Tryptophan | C11H12N2O2 | 203.0819 | 2.5379 | 96.8 | neg | 14635.51199 |
| 434 | 3-Cyano-L-alanine | C4H6N2O2 | 113.0343 | 0.5542 | 41.1 | neg | 14535.85101 |
| 435 | Indene | C9H8 | 117.0701 | 4.3195 | 92.5 | pos | 14524.67901 |
| 436 | Benzofuran | C8H6O | 119.0494 | 3.3773 | 82.5 | pos | 14500.85776 |
| 437 | 2-Methoxy-4-vinylphenol | C9H10O2 | 149.0597 | 2.2806 | 87.2 | neg | 14432.19373 |
| 438 | Terbutaline | C12H19NO3 | 226.1435 | 4.3195 | 37.4 | pos | 14402.88413 |
| 439 | L-Ribose | C5H10O5 | 149.0444 | 3.1446 | 47.9 | neg | 14372.90518 |
| 440 | Cordycepin | C10H13N5O3 | 252.1085 | 0.9699 | 82.5 | pos | 14165.27142 |
| 441 | 3,4-Bis(methoxycarbonyl)benzoic acid | C11H10O6 | 237.04 | 3.9516 | 90.3 | neg | 14018.4918 |
| 442 | Eriodictyol-7-O-glucoside | C21H22O11 | 451.123 | 4.1758 | 82.4 | pos | 13990.01798 |
| 443 | Wighteone | C20H18O5 | 337.1079 | 11.6966 | 55.3 | neg | 13952.97841 |
| 444 | 5,7,3'-Trihydroxy-4'-methoxyflavanone | C16H14O6 | 301.0716 | 6.8189 | 36.2 | neg | 13806.12748 |
| 445 | Morusin | C25H24O6 | 421.1634 | 13.192 | 97.7 | pos | 13688.7619 |
| 446 | 4-Methoxysalicylic acid | C8H8O4 | 151.0389 | 2.9288 | 39.8 | pos | 13465.47885 |
| 447 | Curcolone | C15H18O3 | 291.1236 | 10.1996 | 39.1 | neg | 13384.52504 |
| 448 | (S)-(-)-Perillyl alcohol | C10H16O | 135.1167 | 13.1696 | 93.2 | pos | 13163.08336 |
| 449 | Acetosyringone | C10H12O4 | 195.0653 | 3.9729 | 36.2 | neg | 13089.8353 |
| 450 | (6E)-8-Oxogeraniol | C10H16O2 | 169.1221 | 12.9479 | 67.2 | pos | 13050.01242 |
| 451 | Oxaloacetate | C4H4O5 | 130.9973 | 0.8162 | 41.8 | neg | 12984.33571 |
| 452 | Diisobutyl phthalate | C16H22O4 | 279.1568 | 8.9125 | 39.3 | pos | 12982.16812 |
| 453 | Pinoresinol diglucoside | C32H42O16 | 700.2804 | 3.6137 | 66.5 | pos | 12960.78601 |
| 454 | L-(-)-3-Phenyllactic acid | C9H10O3 | 165.0547 | 4.0351 | 85.8 | neg | 12854.77857 |
| 455 | Inositol | C6H12O6 | 225.0609 | 0.5542 | 81.3 | neg | 12808.25706 |
| 456 | Eriodictyol | C15H12O6 | 289.0703 | 4.4691 | 76.6 | pos | 12740.75058 |
| 457 | Alpha-Calacorene | C15H20 | 201.1635 | 12.926 | 68.1 | pos | 12670.35633 |
| 458 | (-)-Fenchone | C10H16O | 135.1168 | 7.3538 | 45.8 | pos | 12568.22692 |
| 459 | Ginkgolic acid | C20H32O3 | 365.2327 | 12.4968 | 37.7 | neg | 12522.63358 |
| 460 | Uracil | C4H4N2O2 | 113.0349 | 1.4275 | 64.5 | pos | 12426.77128 |
| 461 | (S)-tropic acid | C9H10O3 | 167.0701 | 0.2797 | 63.5 | pos | 12367.04001 |
| 462 | (R)-ar-Turmerone | C15H20O | 217.1585 | 12.7273 | 39.6 | pos | 12196.10918 |
| 463 | Caryophyllene epoxide | C15H24O | 221.1897 | 11.3414 | 86.5 | pos | 12010.4695 |
| 464 | Indole-3-aldehyde | C9H7NO | 146.06 | 2.5709 | 95 | pos | 11970.41861 |
| 465 | Ornithine | C5H12N2O2 | 133.0971 | 0.4887 | 79.8 | pos | 11743.07161 |
| 466 | Myristoleic acid | C14H26O2 | 285.207 | 11.1876 | 54.3 | neg | 11674.70438 |
| 467 | Atractylenolide II | C15H20O2 | 233.1532 | 12.5281 | 96.9 | pos | 11583.14445 |
| 468 | Sphingosine | C18H37NO2 | 300.2892 | 12.4194 | 87.6 | pos | 11275.6161 |
| 469 | Gingerol | C17H26O4 | 293.1757 | 10.7034 | 94.3 | neg | 11254.04644 |
| 470 | Salicylaldehyde | C7H6O2 | 123.0442 | 0.8988 | 62.3 | pos | 11240.77673 |
| 471 | Thymidine | C10H14N2O5 | 241.0826 | 1.8401 | 51.4 | neg | 10853.35515 |
| 472 | Gamma-Glu-Leu | C11H20N2O5 | 261.1442 | 2.7991 | 88 | pos | 10617.24981 |
| 473 | D-Cathine | C9H13NO | 152.1069 | 9.8385 | 81.5 | pos | 10617.03193 |
| 474 | Scytalone | C10H10O4 | 177.0545 | 2.7603 | 70.4 | pos | 10591.5008 |
| 475 | (+)-7-epi-Syringaresinol 4'-glucoside | C28H36O13 | 579.2078 | 4.4717 | 97.8 | neg | 10371.70025 |
| 476 | Scopolamine hydrobromide | C17H21NO4 | 304.154 | 3.0606 | 90.6 | pos | 10252.19016 |
| 477 | Sucrose | C12H22O11 | 341.1086 | 15.8676 | 86.1 | neg | 10155.13778 |
| 478 | 9-Octadecenamide | C18H35NO | 282.2786 | 14.1067 | 97 | pos | 10152.00571 |
| 479 | Phenethyl caffeate | C17H16O4 | 283.0973 | 10.6172 | 81.3 | neg | 10051.84464 |
| 480 | Dihydrooroxylin | C16H14O5 | 285.0767 | 8.2477 | 65.5 | neg | 9748.817614 |
| 481 | 3-methylglutaric acid | C6H10O4 | 145.0495 | 2.4524 | 65.1 | neg | 9602.769597 |
| 482 | Germacrone | C15H22O | 219.174 | 13.3254 | 91.7 | pos | 9572.114987 |
| 483 | Xestodecalactone A | C14H16O5 | 265.1067 | 3.7542 | 39.6 | pos | 9571.07728 |
| 484 | Piperine | C17H19NO3 | 286.1434 | 10.8341 | 96 | pos | 9564.210378 |
| 485 | 4-Ethylphenol | C8H10O | 123.0807 | 3.6543 | 67.8 | pos | 9491.743909 |
| 486 | 2'-Deoxymugineic acid | C12H20N2O7 | 305.1338 | 0.6321 | 42.1 | pos | 9195.363094 |
| 487 | Methyl geranate | C11H18O2 | 183.1379 | 4.4915 | 40.5 | pos | 9009.270015 |
| 488 | Incensole | C20H34O2 | 307.2625 | 13.4839 | 49.3 | pos | 8960.267799 |
| 489 | (+)-Anwulignan | C20H24O4 | 373.1628 | 10.5966 | 56.3 | neg | 8712.884395 |
| 490 | M-Xylene | C8H10 | 107.0859 | 8.3216 | 75.6 | pos | 8702.149798 |
| 491 | 3-Hydroxy-2-Methylpyridine | C6H7NO | 110.0604 | 15.8934 | 39.1 | pos | 8629.105689 |
| 492 | 3-Butylidenephthalide | C12H12O2 | 189.0909 | 6.9942 | 68.5 | pos | 8424.083639 |
| 493 | 3-Methylbenzaldehyde | C8H8O | 121.065 | 2.457 | 62.5 | pos | 8211.927297 |
| 494 | 4-acetoxyphenol | C8H8O3 | 151.039 | 3.0219 | 59.1 | neg | 8078.662295 |
| 495 | Dihydronepetalactone | C10H16O2 | 169.1222 | 5.9002 | 53 | pos | 7971.337759 |
| 496 | Trans-3-Hydroxycinnamate | C9H8O3 | 165.0545 | 0.2797 | 53.6 | pos | 7747.153396 |
| 497 | Cnidioside A | C17H20O9 | 367.1032 | 3.0822 | 41.6 | neg | 7745.352005 |
| 498 | 2-Chromanone | C9H8O2 | 147.044 | 2.7725 | 50.5 | neg | 7609.937699 |
| 499 | 1-Methoxynaphthalene | C11H10O | 159.0803 | 10.6145 | 76.3 | pos | 7566.664895 |
| 500 | 2,6-Dimethylnaphthalene | C12H12 | 157.1011 | 6.6574 | 58.1 | pos | 7467.070209 |
| 501 | Costunolide | C15H20O2 | 233.1534 | 2.7414 | 74.9 | pos | 7366.717302 |
| 502 | Apigenin | C15H10O5 | 269.0454 | 6.5531 | 79.5 | neg | 7341.92257 |
| 503 | Narchinol A | C12H14O3 | 207.1014 | 8.8236 | 63.4 | pos | 7299.127903 |
| 504 | Arteannuin | C15H20O3 | 231.1376 | 0.3663 | 71.5 | pos | 7169.758832 |
| 505 | Pelletierine | C8H15NO | 142.1226 | 3.7338 | 70.3 | pos | 7133.572246 |
| 506 | O-Xylene | C8H10 | 107.0859 | 7.42 | 67.6 | pos | 6987.539442 |
| 507 | Isoeugenol | C10H12O2 | 163.0754 | 6.1152 | 86.9 | neg | 6860.14899 |
| 508 | 2,6-Dihydroxypurine | C5H4N4O2 | 151.025 | 0.8365 | 85.3 | neg | 6709.494091 |
| 509 | 3-Ethyltoluene | C9H12 | 121.1013 | 8.3216 | 80.5 | pos | 6604.792562 |
| 510 | Epinepetalactone | C10H14O2 | 167.1065 | 11.122 | 67.2 | pos | 6591.715008 |
| 511 | Isofraxidin | C11H10O5 | 223.0598 | 4.4915 | 95.6 | pos | 6570.664551 |
| 512 | Indole | C8H7N | 118.0654 | 2.6278 | 67.5 | pos | 6569.048866 |
| 513 | Atropine | C17H23NO3 | 290.1747 | 3.6337 | 86.9 | pos | 6532.944906 |
| 514 | 6-Methylcoumarin | C10H8O2 | 161.0596 | 7.9945 | 64.6 | pos | 6513.685899 |
| 515 | Thymol | C10H14O | 133.1012 | 7.42 | 40.6 | pos | 6468.440619 |
| 516 | Chrysoeriol | C16H12O6 | 299.0558 | 6.951 | 98.2 | neg | 6426.659743 |
| 517 | 10-hydroxy-2E-decenoic acid | C10H18O3 | 187.1327 | 5.9002 | 78.3 | pos | 6211.176936 |
| 518 | Sphinganine | C18H39NO2 | 302.3048 | 12.2244 | 59.2 | pos | 6147.207583 |
| 519 | Alpha,4-Dimethylstyrene | C10H12 | 133.1012 | 5.4096 | 89.8 | pos | 6140.988297 |
| 520 | Andrographolide | C20H30O5 | 395.2049 | 11.0555 | 46 | neg | 6135.246927 |
| 521 | L-Valine | C5H11NO2 | 118.0865 | 15.3966 | 68.8 | pos | 6105.665812 |
| 522 | Eremophila-1(10),8,11-triene | C15H22 | 203.1792 | 13.1037 | 86.2 | pos | 6102.232223 |
| 523 | 6-methyluracil | C5H6N2O2 | 127.0502 | 1.82 | 83.4 | pos | 5988.718907 |
| 524 | 5-Methyl-2-furaldehyde | C6H6O2 | 111.0444 | 5.2332 | 69.7 | pos | 5962.138361 |
| 525 | Sinapyl aldehyde | C11H12O4 | 207.0656 | 4.6221 | 95.4 | neg | 5879.003457 |
| 526 | L-Tyrosine | C9H11NO3 | 182.081 | 0.6677 | 95 | pos | 5727.767176 |
| 527 | Veratric acid | C9H10O4 | 183.065 | 15.9376 | 38.8 | pos | 5472.352419 |
| 528 | Curcumenol | C15H22O2 | 235.1689 | 3.3952 | 83.2 | pos | 5373.175153 |
| 529 | 3-Methyl-2-oxobutanoic acid | C5H8O3 | 117.0549 | 0.2797 | 46.2 | pos | 5371.056918 |
| 530 | Sinapic acid | C11H12O5 | 223.0606 | 3.8469 | 97.7 | neg | 5361.409885 |
| 531 | Chamazulene | C14H16 | 185.1323 | 13.1037 | 87.8 | pos | 5282.873755 |
| 532 | Hexadecanedioic acid | C16H30O4 | 285.2069 | 12.6263 | 89.6 | neg | 5219.899059 |
| 533 | Delta-Hexalactone | C6H10O2 | 115.0756 | 9.1817 | 55.9 | pos | 5007.028857 |
| 534 | Dillapiole | C12H14O4 | 221.0813 | 8.6854 | 42.9 | neg | 4846.472435 |
| 535 | Safflomin A | C27H32O16 | 630.2021 | 3.5138 | 40.2 | pos | 4782.074768 |
| 536 | (-)-Salsolinol | C10H13NO2 | 162.0912 | 4.1375 | 72.4 | pos | 4692.508554 |
| 537 | Gamma-GLU-PHE | C14H18N2O5 | 295.1286 | 3.004 | 82.5 | pos | 4435.293212 |
| 538 | Diglykokoll | C4H7NO4 | 134.0447 | 0.6144 | 46.2 | pos | 4418.565046 |
| 539 | Hesperidin | C28H34O15 | 611.1901 | 9.622 | 38.2 | pos | 4353.190078 |
| 540 | Linolenic acid ethyl ester | C20H34O2 | 307.2627 | 13.8789 | 69.2 | pos | 4099.435342 |
| 541 | Fraxinol | C11H10O5 | 223.0598 | 4.1962 | 76.5 | pos | 3757.966808 |
| 542 | Pteroside A | C21H30O8 | 411.2005 | 4.278 | 62.5 | pos | 3753.675855 |
| 543 | (2R)-and (2S)-eriodictyol-7-O-beta-D-glucopyranoside | C21H22O11 | 451.1229 | 3.5138 | 85.1 | pos | 3721.574806 |
| 544 | Carnitine | C7H15NO3 | 162.1123 | 15.3966 | 38.3 | pos | 3548.445012 |
| 545 | P-Tolualdehyde | C8H8O | 121.0649 | 10.6361 | 80.4 | pos | 3534.230017 |
| 546 | 2,3-Butanediol | C4H10O2 | 73.0655 | 1.0769 | 58.4 | pos | 3522.928102 |
| 547 | Pterosin G | C14H18O3 | 235.1326 | 6.1678 | 73.1 | pos | 3503.941232 |
| 548 | Farnesyl acetone | C18H30O | 280.263 | 13.8415 | 93.1 | pos | 3380.848122 |
| 549 | Styrene | C8H8 | 105.0702 | 12.5281 | 97.6 | pos | 3317.156947 |
| 550 | Lumichrome | C12H10N4O2 | 243.0874 | 4.6667 | 92.8 | pos | 3316.463586 |
| 551 | Parthenin | C15H18O4 | 263.1268 | 8.8236 | 60.9 | pos | 3272.119249 |
| 552 | Citrulline | C6H13N3O3 | 174.0873 | 0.5729 | 70.8 | neg | 3250.656138 |
| 553 | Trans,trans-2,4-Heptadienal | C7H10O | 111.0808 | 11.5173 | 60.6 | pos | 3247.764257 |
| 554 | 1,4-Dicaffeoylquinic acid | C25H24O12 | 499.1227 | 4.3608 | 73.7 | pos | 3211.659831 |
| 555 | 6-Paradol | C17H26O3 | 261.1846 | 9.2922 | 45.9 | pos | 3123.672945 |
| 556 | 6-beta-D-Glucopyranosyl-8-beta-D-ribopyranosylapigenin | C26H28O14 | 563.1402 | 3.556 | 93 | neg | 3008.702675 |
| 557 | 4-Hydroxycinnamamide | C9H9NO2 | 181.097 | 0.9521 | 36.3 | pos | 2992.734751 |
| 558 | (-)-Syringaresinol di-O-glucoside | C34H46O18 | 787.2654 | 3.745 | 96.8 | neg | 2966.238669 |
| 559 | Nicotianamine | C12H21N3O6 | 302.1356 | 1.6679 | 64.4 | neg | 2864.969786 |
| 560 | 4,7-Dimethyl-1-tetralone | C12H14O | 175.1118 | 3.8319 | 63.5 | pos | 2795.635506 |
| 561 | Cadalene | C15H18 | 199.1479 | 3.8319 | 62.7 | pos | 2743.15825 |
| 562 | Atractylenolide III | C15H20O3 | 231.1377 | 10.8341 | 94.2 | pos | 2725.499643 |
| 563 | Methylhydroquinone | C7H8O2 | 125.0598 | 6.1897 | 65.9 | pos | 2692.935734 |
| 564 | Palmitoyl Ethanolamide | C18H37NO2 | 300.2893 | 13.8897 | 57.9 | pos | 2643.858269 |
| 565 | Arglabin | C15H18O3 | 247.1325 | 10.7902 | 75.4 | pos | 2597.372378 |
| 566 | 4-Pyridinol | C5H5NO | 96.0449 | 15.9156 | 38.6 | pos | 2424.129335 |
| 567 | Norharman | C11H8N2 | 169.076 | 3.4744 | 52.3 | pos | 2276.603928 |
| 568 | Stemofuran A | C14H10O3 | 227.0699 | 2.7792 | 64.8 | pos | 2234.376389 |
| 569 | Brazilin | C16H14O5 | 287.0911 | 2.7792 | 67.5 | pos | 2175.353453 |
| 570 | 7-Isopropyl-1,4-dimethylazulene | C15H18 | 199.1479 | 12.071 | 87 | pos | 2154.373217 |
| 571 | 1,3-O-Dicaffeoylquinic acid | C25H24O12 | 515.1198 | 3.4335 | 96.8 | neg | 2059.258104 |
| 572 | 4-Methoxycinnamic acid | C10H10O3 | 161.0596 | 2.7792 | 68 | pos | 2042.98357 |
| 573 | Dehydroabietic acid | C20H28O2 | 301.2157 | 12.071 | 70.8 | pos | 2037.80686 |
| 574 | Nistose | C24H42O21 | 665.214 | 0.7963 | 98.4 | neg | 1986.406074 |
| 575 | Furanodienon | C15H18O2 | 231.1376 | 9.0919 | 70.7 | pos | 1927.528641 |
| 576 | Atractyloside G | C21H36O8 | 461.2393 | 3.9729 | 66.1 | neg | 1891.891822 |
| 577 | 3,3',5-Trimethoxybibenzyl | C17H20O3 | 331.1546 | 13.4258 | 41.8 | neg | 1873.591282 |
| 578 | 2-buten-1-one, 1-(2,6,6-trimethyl-1,3-cyclohexadien-1-yl)- | C13H18O | 191.1429 | 2.7414 | 77.5 | pos | 1837.693044 |
| 579 | [8]-Shogaol | C19H28O3 | 305.2107 | 13.2584 | 61.6 | pos | 1810.071128 |
| 580 | 6-Methyl indole | C9H9N | 132.0808 | 2.5709 | 85.5 | pos | 1775.060489 |
| 581 | Linolenyl alcohol | C18H32O | 282.2787 | 14.8418 | 85.3 | pos | 1765.11298 |
| 582 | Indoleacetic acid | C10H9NO2 | 176.0705 | 4.0769 | 68.3 | pos | 1751.967664 |
| 583 | Forskolin | C22H34O7 | 411.2358 | 6.9715 | 36.5 | pos | 1738.84058 |
| 584 | Dehydro-1,8-cineole | C10H16O | 151.1118 | 6.8623 | 82.6 | neg | 1732.587424 |
| 585 | Vulgarin | C15H20O4 | 265.1431 | 7.8593 | 63.5 | pos | 1604.979692 |
| 586 | Curdione | C15H24O2 | 237.1845 | 6.9489 | 78.3 | pos | 1503.811741 |
| 587 | Myristic acid | C14H28O2 | 246.2424 | 7.7687 | 52.5 | pos | 1487.551146 |
| 588 | 4-Methyl-5-thiazoleethanol | C6H9NOS | 144.0477 | 1.5252 | 53.4 | pos | 1487.122623 |
| 589 | Carnosol | C20H26O4 | 375.1787 | 12.2818 | 67.1 | neg | 1409.809835 |
| 590 | Turmeronol B | C15H20O2 | 233.1534 | 3.913 | 46.3 | pos | 1356.992647 |
| 591 | Soyasapogenol C | C30H48O2 | 423.3611 | 14.208 | 38.4 | pos | 1270.473521 |
| 592 | Rubescensin A | C20H28O6 | 363.1808 | 8.1154 | 46.2 | neg | 1149.915556 |
| 593 | Pinobanksin | C15H12O5 | 273.0751 | 10.4153 | 61.5 | pos | 975.5746993 |
| 594 | Oxypeucedanin hydrate | C16H16O6 | 305.1016 | 5.7222 | 97.8 | pos | 970.1777073 |
| 595 | 13Z-Docosenamide | C22H43NO | 338.341 | 15.2388 | 77.2 | pos | 943.6482384 |
| 596 | Coniferyl alcohol | C10H12O3 | 163.0752 | 10.8341 | 40 | pos | 867.4871596 |
| 597 | 7-Demethylsuberosin | C14H14O3 | 229.0864 | 10.3987 | 90.7 | neg | 841.5413898 |
| 598 | L-Histidine | C6H9N3O2 | 156.0766 | 2.3822 | 89.9 | pos | 823.4654504 |
| 599 | Xanthotoxol | C11H6O4 | 203.0337 | 11.7168 | 90.3 | pos | 813.4518697 |
| 600 | Methyl linolenate | C19H32O2 | 293.2469 | 13.6923 | 62.9 | pos | 811.8670627 |
| 601 | Actidione | C15H23NO4 | 264.159 | 6.7683 | 76 | pos | 800.7297237 |
| 602 | Homoorientin | C21H20O11 | 447.0931 | 3.5987 | 87.8 | neg | 702.8320248 |
| 603 | Byakangelicol | C17H16O6 | 317.1015 | 5.9673 | 91.4 | pos | 697.93783 |
| 604 | Alpha-Corocalene | C15H20 | 201.1635 | 13.3254 | 82.6 | pos | 688.4561194 |
| 605 | 7-Hydroxycadalene | C15H18O | 215.1427 | 2.7414 | 37.1 | pos | 661.0431586 |
| 606 | H-ile-ile-oh | C12H24N2O3 | 243.171 | 3.0412 | 62.1 | neg | 604.719367 |
| 607 | Lycoperodine 1 | C12H12N2O2 | 215.082 | 3.1446 | 90.7 | neg | 577.3307573 |
| 608 | Anthracene | C14H10 | 179.0853 | 12.6392 | 93.1 | pos | 572.0028756 |
| 609 | H-Glu-Tyr-OH | C14H18N2O6 | 291.0986 | 2.6464 | 52.8 | neg | 548.3039811 |
| 610 | Feruloylputrescine | C14H20N2O3 | 263.1399 | 3.2055 | 86.8 | neg | 540.2165641 |
| 611 | Kainic acid | C10H15NO4 | 212.0922 | 3.8059 | 57.4 | neg | 525.2097659 |
| 612 | Alpha-Farnesene | C15H24 | 205.1948 | 13.1696 | 62 | pos | 481.6529906 |
| 613 | Palmitic amide | C16H33NO | 256.263 | 14.0499 | 40.6 | pos | 468.3862898 |
| 614 | (+)-alpha-Curcumene | C15H22 | 203.1792 | 14.6719 | 89 | pos | 447.3006256 |
| 615 | Longifolenaldehyde | C15H24O | 221.1898 | 9.8167 | 83.7 | pos | 415.2340254 |
| 616 | Alpinumisoflavone | C20H16O5 | 337.1063 | 12.572 | 62.5 | pos | 397.4850834 |
| 617 | Vitexin | C21H20O10 | 431.098 | 3.9729 | 89.2 | neg | 375.626058 |
| 618 | Tryptamine | C10H12N2 | 161.1073 | 2.9288 | 94 | pos | 372.028452 |
| 619 | Beta-Calacorene | C15H20 | 201.1635 | 13.6139 | 85.3 | pos | 369.6356013 |
| 620 | Androstenedione | C19H26O2 | 328.2265 | 13.4611 | 35.1 | pos | 369.0469327 |
| 621 | Pterosin Z | C15H20O2 | 274.1796 | 12.5281 | 78.4 | pos | 333.3084451 |
| 622 | Flemiphilippinin A | C30H32O6 | 489.2263 | 13.5291 | 75.2 | pos | 322.6086915 |
| 623 | Aristolene | C15H24 | 205.1949 | 12.9696 | 79.5 | pos | 264.0780464 |
| 624 | Dehydrocostus lactone | C15H18O2 | 231.1377 | 14.433 | 84.5 | pos | 263.2124782 |
| 625 | Pterosin B | C14H18O2 | 183.1166 | 13.1696 | 51.8 | pos | 243.2034849 |
| 626 | Acenaphthene | C12H10 | 155.0853 | 12.6392 | 91.2 | pos | 243.1851753 |
| 627 | Glabrone | C20H16O5 | 335.0923 | 11.5407 | 53.5 | neg | 202.7860465 |
| 628 | L-Leucine | C6H13NO2 | 173.1285 | 2.8559 | 41.7 | pos | 163.8724352 |
| 629 | Dihydrojasmonic acid, methyl ester | C13H22O3 | 227.1638 | 13.236 | 56.8 | pos | 160.9130628 |
| 630 | Atalantoflavone | C20H16O5 | 335.0922 | 12.5833 | 82.9 | neg | 138.5304059 |
| 631 | N-Phenyl-1-naphthylamine | C16H13N | 220.1117 | 12.6392 | 49.2 | pos | 127.4701802 |
| 632 | Ponicidin/Rubescensin B | C20H26O6 | 363.1773 | 4.4467 | 64.7 | pos | 95.74676181 |
| 633 | Dictamnoside I | C21H38O9 | 479.2496 | 2.8973 | 93.9 | neg | 86.87876504 |
| 634 | Nardosinone | C15H22O3 | 215.1428 | 13.5521 | 36.1 | pos | 85.44781213 |
| 635 | Parthenolide | C15H20O3 | 231.1377 | 13.0815 | 79.4 | pos | 68.71968582 |
| 636 | (+)-longifolene | C15H24 | 205.195 | 5.9898 | 70.8 | pos | 68.31425425 |
| 637 | Gamma-Calacorene | C15H20 | 201.1635 | 13.8217 | 93.4 | pos | 64.73260714 |
| 638 | Atractylenolide I | C15H18O2 | 248.1642 | 8.8012 | 84.7 | pos | 56.47351551 |
| 639 | 5,6-Dehydrokawain | C14H12O3 | 227.0708 | 10.944 | 46.3 | neg | 36.4815498 |
| 640 | Triptophenolide | C20H24O3 | 330.2058 | 9.8167 | 59.2 | pos | 33.89553343 |
| 641 | Alpha-Cyperone | C15H22O | 236.2006 | 5.6772 | 74.7 | pos | 30.86040738 |
| 642 | Linderalactone | C15H16O3 | 245.117 | 8.8236 | 57.8 | pos | 22.40387988 |
| 643 | L-Stepholidine | C19H21NO4 | 345.1802 | 9.4236 | 69.1 | pos | 15.80578864 |
| 644 | Isoalantolactone | C15H20O2 | 233.1533 | 14.0499 | 90.7 | pos | 15.40756623 |
| 645 | 1-Naphthylmethanol | C11H10O | 141.0697 | 10.8341 | 65.9 | pos | 11.45982471 |
| 646 | Trans-Stilbene | C14H12 | 181.1009 | 13.1264 | 81.4 | pos | 0.964722656 |
