# Supplementary material for: Identification of the immunoprotective activity of Mori Cortex Radicis ethanol extract against aquaculture pathogens (Aeromonas veronii, Pseudomonas aeruginosa)
Source: Front Immunol. 2026 Aug 17;17:1927434. doi: 10.3389/fimmu.2026.1927434 (PMC13526930; doi:10.3389/fimmu.2026.1927434)
Supplement: Supplementary file 1 [file DataSheet1.zip › Supplementary files/Supplementary Table S3.docx]

**Supplementary Table S3** Analysis of MCR-EE feeding concentrations following *A. veronii* challenge.

| **Concentrations (mg/kg)** | **Fish**  **(No.)** | **Death**  **(no.)** | **Survival**  **(no.)** | **ADR**  **(%)** | **RPS**  **(%)** |
| --- | --- | --- | --- | --- | --- |
| 100 | 10 | 7 | 3 | 70 | 30 |
| 200 | 10 | 4 | 6 | 40 | 60* |
| 400 | 10 | 6 | 4 | 60 | 40 |
| Control (0) | 10 | 10 | 0 | 100 | -- |

* *p* < 0.05 (compared with control). ADR: accumulated death rate. RPS: relative percent survival; RPS (%) = [1 − (% experimental group mortality/% control mortality)] × 100.
